# *Cynanchum auriculatum* Royle ex Wight., *Cynanchum bungei* Decne. and *Cynanchum wilfordii* (Maxim.) Hemsl.: Current Research and Prospects

**DOI:** 10.3390/molecules26237065

**Published:** 2021-11-23

**Authors:** Lu Wang, Fujie Cai, Wei Zhao, Jinli Tian, Degang Kong, Xiaohui Sun, Qing Liu, Yueru Chen, Ying An, Fulin Wang, Xue Liu, Yi Wu, Honglei Zhou

**Affiliations:** 1 College of Pharmacy, Shandong University of Traditional Chinese Medicine, Jinan 250355, China; superlu777999@163.com (L.W.); Caifj1234@163.com (F.C.); w17862951781@163.com (W.Z.); tianjinli1998@163.com (J.T.); magicalgang@163.com (D.K.); sun_xiaohui@126.com (X.S.); Lq9775725@163.com (Q.L.); cyr1049559713@163.com (Y.C.); anying323@163.com (Y.A.); qingdainanxing@163.com (F.W.); lxlsh24844284@163.com (X.L.); 2 Institute of Traditional Chinese Veterinary Medicine, College of Veterinary Medicine, Nanjing Agricultural University, Nanjing 210095, China; wuyi2001cn@njau.edu.cn

**Keywords:** *Cynanchum* *auriculatum* Royle ex Wight., *Cynanchum bungei* Decne., *Cynanchum wilfordii* (Maxim.) Hemsl., caudatin, cynandione A

## Abstract

*Cynanchum auriculatum* Royle ex Wight. (*CA*), *Cynanchum bungei* Decne. (*CB*) and *Cynanchum wilfordii* (Maxim.) Hemsl. (*CW*) are three close species belonging to the Asclepiadaceous family, and their dry roots as the bioactive part have been revealed to exhibit anti-tumor, neuroprotection, organ protection, reducing liver lipid and blood lipid, immunomodulatory, anti-inflammatory, and other activities. Until 2021, phytochemistry investigations have uncovered 232 compounds isolated from three species, which could be classified into C_21_-steroids, acetophenones, terpenoids, and alkaloids. In this review, the morphology characteristics, species identification, and the relationship of botany, extraction, and the separation of chemical constituents, along with the molecular mechanism and pharmacokinetics of bioactive constituents of three species, are summarized for the first time, and their phytochemistry, pharmacology, and clinical safety are also updated. Moreover, the direction and limitation of current research on three species is also discussed.

## 1. Introduction

*Cynanchum auriculatum* Royle ex Wight. (*CA*), *Cynanchum bungei* Decne. (*CB*) and *Cynanchum wilfordii* (Maxim.) Hemsl. (*CW*) are three related species, commonly known as “Baishouwu”, and locals also call *CA* “Binhai baishouwu”, *CB* “Taishan baishouwu”, as well as *CW* “Paeksuo or Paekhasuo” [1,2,3]. Among them, *CA* and *CW* are mainly distributed in China, Japan, and Korea; while *CB* is concentrated in China, such as Shandong, Henan, Hebei, and Gansu provinces [4,5]. Current study has unraveled that *CA*, *CB* and *CW* displayed high medicinal value, exactly as C_21_-steroids were found to exhibit strong anti-tumor activity mainly involving hepatoma, breast cancer, and human glioma; while acetophenones and crude extracts of three species were revealed to exert immunomodulatory, hepatoprotection, anti-inflammatory, and anti-depressant activities [6]. Moreover, planting *CA* was able to bring high economic benefits, as it could be processed into flower tea and starch to be applied to the food industry [7].

There is a long history of medicinal use for the three species. *CB* was firstly recorded in the “Heshouwu Biography” from the Tang Dynasty, which explained that the red was a male called “Heshouwu”, the white was a female called “Baishouwu” [8]. Additionally, the nourishing blood, hepatoprotection, and strengthening kidney effects of *CW* were firstly recorded in the “Compendium of Herbology” in Ming Dynasty [9]. At present, *CA*, *CB*, and *CW* have been served as traditional medicinal plants in China, Japan, and Korea, and they have been registered in “New Chinese Medicinal Herbal” [6]. Furthermore, a gas chromatography-mass spectrometer analysis revealed that there were different chemical constituents and biological activities in three species [10]. However, in traditional use, the phenomenon of mixing three species and unprecise pharmacology records in classic books were common due to the lack of scientific data at that time. Intriguingly, current study has addressed the above issues through several experiments, which contributed to helping us to apply them scientifically. It is worth noting that their application is not limited to individual species; as a matter of fact, they could be available to combine with other herbs to effectively treat common diseases. For instance, *CW* combined with *Arctium lappa* L. (Compositae) and *Dioscorea opposite* Thunb (Dioscoreaceae) were revealed to exhibit a potent suppressive effect on vascular and hepatic inflammation in mice fed a high fructose diet [11].

According to the published references, botany, phytochemistry, pharmacology, molecular mechanism, and the pharmacokinetics of bioactive constituents, as well as the clinical safety of *CA*, *CB* and *CW*, were compiled comprehensively in this review. Furthermore, we also proposed some suggestions regarding the limitation of three species study, aiming to provide a reference for relevant researchers.

## 2. Botany

### 2.1. Morphological Characteristics

**Figure 1:**
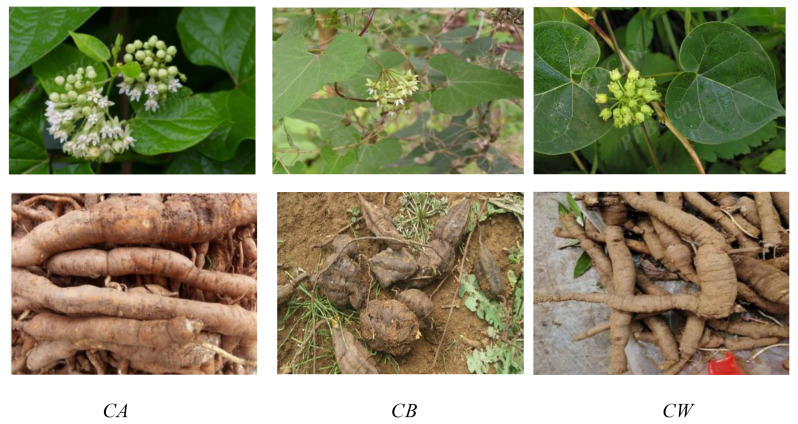
Morphological characteristics of *CA*, *CB* and *CW*.

*CA*: The roots are plump, and appear cylindrical and tuberous with a brownish-yellow surface. Its stems and leaves are covered with microscopic hairs, and leaves are oval, 4–12 cm long, and 4–10 cm wide. There are about 30 flowers with a soft hairy inner surface in individual, appearing white and oval-shaped. Moreover, its follicles double in lanceolate with 8 cm long and 1 cm in diameter. Normally, its flowering period is 6–9 months, and the fruit period is 7–11 months. *CB*: The roots are cylindrical or irregular masses with the brown-white surface, existing mostly 3–6 in number. The stems are slender with micro-hairs, and the leaves are halberd-shaped, 3–8 cm long, and 1–5 cm wide at the base. The calyx lobes are needle-shaped, and the corolla appears white or yellow-green with a soft hairy inner surface. The seed apex has numerous white filaments about 4 cm long. Its flowering period is 6–7 months, and its fruit period is 7–10 months. *CW*: The roots are spindle shaped and gray-brown, appearing about 10 cm long and 2 cm in diameter. The stems are slender with soft hair to cover, and leaves are ovate, 5–6 cm long, and 2–4 cm wide. There are about 15–20 flowers in the individual with soft hairs outside the calyx, and the corolla is yellowish, showing an oblong shape. Its follicles are single-born and needle-shaped, 12 cm long, and 1 cm in diameter. The seed is egg-shaped with a dark brown surface. Its flowering period is 5–9 months, and its fruit period is 7–10 months. The morphological characteristics of *CA*, *CB* and *CW* are displayed in Figure 1.

### 2.2. Identification of CA, CB and CW

A survey reported that there was a serious phenomenon to mix among *CA*, *CB* and *CW* in Korean and Chinese markets due to their similarities in appearance [12]. At present, the methods of chemical analysis and molecular markers have been conducted to differentiate them. The chemical analysis mainly involving UV, IR, HPLC, MS, NMR, and other techniques, is subjected to obtain typical chemical markers or unique information data to distinguish close species. For example, in the genus of *Cynanchum*, axasterol acetate, metaplexigenin, and stigmasterol were only isolated from *CA*, suggesting they could be recognized as chemotaxonomic markers to differentiate *CA* and the other two species [13]. Equally, a study revealed that conduritol F had the potential to be developed into a chemical marker to distinguish *CA* and *CW*, evidenced by its H-5 and H-6 signals being well-separated from other signals by the analysis of quantitative NMR [14]. It is noteworthy that alkaloids isolated from three species were all from *CA*, which could be used as chemical markers to distinguish *CA* from the other two species [13,15,16]. In terms of unique information data, a study unraveled that *CA* and *CW* were available to analyze by the terahertz spectroscopic, leading to *CW* having a higher time delay than *CA*, which indicated it was an efficient method to differentiate related species based on their differences of permittivity [17]. Another study reported that paper cone spray ionization combined with MS contributed to rapidly determining chemical signatures of *CA* and *CW*, which could be efficiently applied to distinguish similar herbs [18]. 

Molecular markers based on inter-individual nucleotide sequence variation that are universally found in the ribosomes and chloroplasts can directly reflect a specific DNA fragment in the genome of an organism or population at the DNA level [19]. They can not only accurately and efficiently differentiate similar species, but also be applied to distinguish pure and heterozygote species [20]. Currently, different primers are combined with PCR enabled specific DNA fragments to amplify, which is conducive to confirming the unique molecular markers of plants. A study reported that unique fragments of *CA* and *CW* combined with PCR were subjected to obtain characterized amplification region markers to distinguish them [21]. Another study revealed that molecular makers of *CA* and *CW* were obtained by their fragments united with conventional polymerase chain reaction PCR and real-time fluorescence quantitative PCR technology [22]. Notably, the appearance of internal transcribed spacer 2 made for reaching identification rates of 90.8% and 87.4% at the species level by BLAST and nearest distance methods, respectively, leading to an effective method to distinguish medicinal plants in the genus *Cynanchum* [23]. Nonetheless, to our knowledge, there was no report regarding molecular markers of *CB* in the previous study, and therefore further investigation was needed to find different molecular markers among *CB* and the other two species. 

### 2.3. Relationship of CA, CB and CW

As we all know, common chemical constituents are often isolated from plants that have close relationships with each other, which can reveal their chemotaxonomic significance to some extent. For example, caudatin, 2,4-dihydroxyacetophenone, 2,5-dihydroxyacetophenone, 4-hydroxyacetophenone, and baishouwubenzophenone were all isolated from *CA*, *CB* and *CW*, indicating there were close relationships among them [24,25,26,27,28,29]. C_21_-steroids (deacylmetaplexigenin, rostratamine, wilfoside C1G, cynanauriculoside E, kidjoranin, cyanoauriculoside A, wilfoside M1N), acetophenones (cynandione A–B, cynandione E), and other compounds (leucanthemitol, suceinie acid, sucrose, methyleugenol) were all obtained from *CA* and *CW*, which might strengthen the relationship between them [5,27,28,30,31,32,33,34,35,36,37,38,39,40,41]. Bungeiside C, bungeiside D and 2-*O*-β-laminaribiosyl-4-hydroxyacetophenone were obtained from *CB* and *CW*, thereby revealing their close relationship between them [42,43]. Consequently, the chemotaxonomic significance of some compounds isolated from *CA*, *CB* and *CW* might support their close relationships, but more evidence was still needed.

Current determination of the relationship among *CA*, *CB*, and *CW* is concentrated on the method of the molecular marker, owing to its precise and scientific characters. Among three species, *CA* and *CW* were revealed to be the most closely related based on the analysis of the reported chloroplast genome [44]. Moreover, *CW* was demonstrated to have a close relationship with *Asclepias nivea* L. (Apocynaceae) and *Asclepias syriaca* L. (Apocynaceae) based on their mitochondrial genome [45,46]. Subsequently, compared with the genome of *Asclepias syriaca* L., *CA* was also demonstrated to be like *Asclepias syriaca* L., revealing their close relationship to some extent [47]. Unfortunately, few species were uncovered to be similar to *CA*, *CB* and *CW*, and therefore more evidence should be demanded to illustrate their close relationships with other herbs, which contributed to discovering their alternatives and related species.

## 3. Phytochemistry

### 3.1. Extraction and Separation

Extraction and separation are the basis of phytochemistry in medicinal plants, and consequently selecting efficient extraction methods and optimizing extraction conditions are of great interest in this direction [48]. Currently, systematic extraction was conducted to unravel chemical constituents of *CA*, *CB* and *CW*, which are characterized by versatility, efficiency, and convenience [4]. The steps are shown as follows: first, their roots are ground into powder to extract, using ethanol or methyl alcohol reflux in an extractor while setting an appropriate time and temperature, to obtain an extracting solution. Second, the extracting solution is extracted with reagents of different polarities, such as petroleum ether, dichloromethane, ethyl acetate, and n-butanol, to yield extracts of different polarities. Third, the extracts are concentrated and separated by elution on silica gel columns combined with reagents of different polarities to obtain different monomer components. Sometime it is necessary to purify different fractions by gel column, microporous resin, and preparative liquid phases. Finally, the chemical structure and formula of monomeric components are determined by UV, IR, MS, and NMR. Strikingly, if we want to obtain polysaccharide components, the roots need to be extracted by water decoction to obtain water extracts, and the subsequent purification operations will be carried out [49]. 

The optimization of the extraction is a key step in the phytochemistry study, which is beneficial to increase the extraction rate of the isolated components by changing the conditions and methods [50]. High-speed counter-current chromatography (HSCC) has been extensively applied to the separation and purification of acetophenones [51]. On the one hand, baishouwubenzophenone, 4-hydroxyacetophenone, 2,4-dihydroxy-acetophenone, and 2,5-dihydroxyacetophenone could be rapidly determined by HSCC, owing to its excellent reproducibility and high-separation efficiency [52]. On the other hand, components with similar structures, low concentration, and high polarity, such as bungeiside-A, bungeiside-B, and baishouwubenzophenone, could also be isolated by HSCC [53]. Furthermore, pressurized liquid extraction combined with a Box-Behnken design optimization was applied to the extraction of *CB*, resulting in it could be recognized as an efficient method to isolate acetophenones [54]. A study established an aqueous ionic liquid comprising three kinds of l-alkyl-3-methylimidazolium, and optimized ultrasound parameters including ionic liquid concentration, solvent to solid ratio, power, particle size, temperature, and extraction time, leading to a high extraction rate of acetophenones [24]. For other compounds, C_21_-steroids of *CA* were optimized to yield 49% content by single-factor orthogonal, and the best extraction conditions were revealed to be 95% ethanol and refluxed twice for 1.5 h each [55]. Polysaccharide yield of *CA* was optimized to reach 1.35% by single-factor with response surface methodology, and the best parameters were displayed as follows: liquid to material ratio was 22:1 mL/g, ultrasonic time was 44 min, ultrasonic power was 350 W, and the ultrasonic temperature was 52 °C [56].

### 3.2. Chemical Constituents

232 compounds have been isolated from *CA*, *CB*, and *CW* as of October 2021, including 171 C_21_-steroids, 28 acetophenones, 12 terpenoids, 6 alkaloids, and 15 others. Of note, C_21_-steroid compounds were the main presences, of which caudatin and its derivatives as primary bioactive constituents have been revealed to exhibit remarkable anti-tumor activity, representing typical chemical and pharmacological features of three species [6]. Besides, acetophenones were the second metabolites in three species, of which cynandione A as the major bioactive compound has been demonstrated to exhibit extensive pharmacological activities, such as neuroprotection and hepatoprotection, which were characterized by healthcare activity [57,58]. Furthermore, reports regarding biological activities of terpenoids, alkaloids, and other constituents were refined, owing to their numbers being limited, which prompted us to explore more chemical constituents in the future.

#### 3.2.1. C_21_-Steroids

It was reported that C_21_-steroid compounds isolated from *CA*, *CB*, and *CW* focused on chloroform and ethyl acetate fractions of crude extracts, and the content of root tuber was higher than root bark [59]. C_21_-steroid compounds are a class of steroid derivatives containing 21 carbon atoms, whose basic backbone is pregnane or their isomer [60]. Caudatin and kidjoranin were primary core families in pregnane glycosides, characterized by the neutral loss of ikemamic acid molecule (128 Da) and cinnamic acid (148 Da) from the precursor ion, respectively [61]. Crystalline and neutral substances of amorphous powder are primary states in C_21_-steroid compounds, with certain optical rotatory, and slightly soluble in water. Pregnane (**I**), seco-pregnane (**II**), trihydroxypregnane (**III**) are representative skeletons in C_21_-steroid compounds of three species, of which skeleton **I** is the main form. In skeleton **I**, A/B and B/C rings are trans-configurations and the C/D ring is the cis configuration. Skeleton **a** and **b** are representative structures in skeleton **I**, of which C10 in skeleton **a** connects with hydrogen, while skeleton **b** does not. At the C3 position, OH connected by monosaccharide, such as 2-deoxy digitalis, diginose, glucose, digitoxose, cymarose, and single OH, are primary to form the C_21_ glycoside [6]. Generally, C5 and C6 are double bond, C8 and C14 links with β-OH, C12 links with β-OH or ester group connected by OH and organic acid, C17 has more α conformation than β in the side chain, and C20 connects with carbonyl and ester group. These patterns are displayed in representative compounds, such as caudatin, caudatin-2,6-dideoxy-3-*O*-methy-β-d-cymaropyranoside, cynanbungeigenin C, aauriculoside A, kidjoranin 3-*O*-β-digitoxopyranoside, gagaminine, qingyangshengenin, wilfoside C3N, wilfoside KIN, cynsaccatol N. However, some peculiar compounds such as metaplexigenin, caudatin-3-*O*-β-cymaropyranoside, 12β-*O*-(4-hydroxybenzoyl)-8β,14β,17β-trihydroxypregn-2,5-diene-20-one, eleutherosidea do not exhibit these patterns. In the skeleton **II,** C8 links with the carbonyl group, C14 links with the carbonyl group or β-OH, C12 connects with the phenyl acrylate, and C20 connects with the acetate group, such as cynabungoside A-C, wilfoside G, and cyanoauriculoside F. In the skeleton **III**, C12 and C14 form epoxy ether and C17 connects with phenyl acrylate in the side chain, such as 17β-*O*-cinnamoyl-3β, 8β,14β-trihydroxypregn-12,20-ether. 

**Figure 2:**
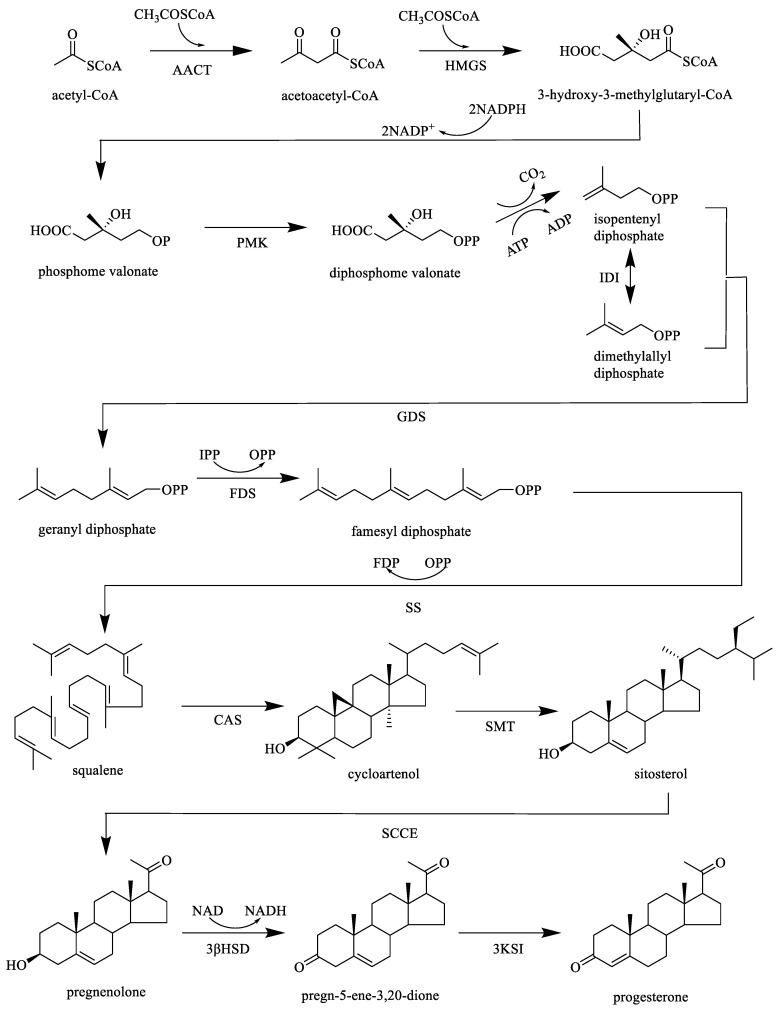
Biosynthesis pathway of C_21_-steroids. (Note: AACT: acetoacetyl-CoA thiolase, HMGS: 3-hydroxy-3-meth-ylglutaryl-CoA synthase, PMK: phosphomevalonate kinase, IDI: isopentenyldiphosphate isomerase, GDS: geranyl diphosphate synthase, FDS: farnesyl diphosphate synthase, SS: squalene synthase, CAS: cycloartenol synthase, SMT: sterol methyltransferase, SCCE: side-chain cleaving enzyme, 3βSD: 3β-hydroxysteroiddehydrogenase, 3KSI: 3-ketosteroidisomerase).

**Figure 3:**
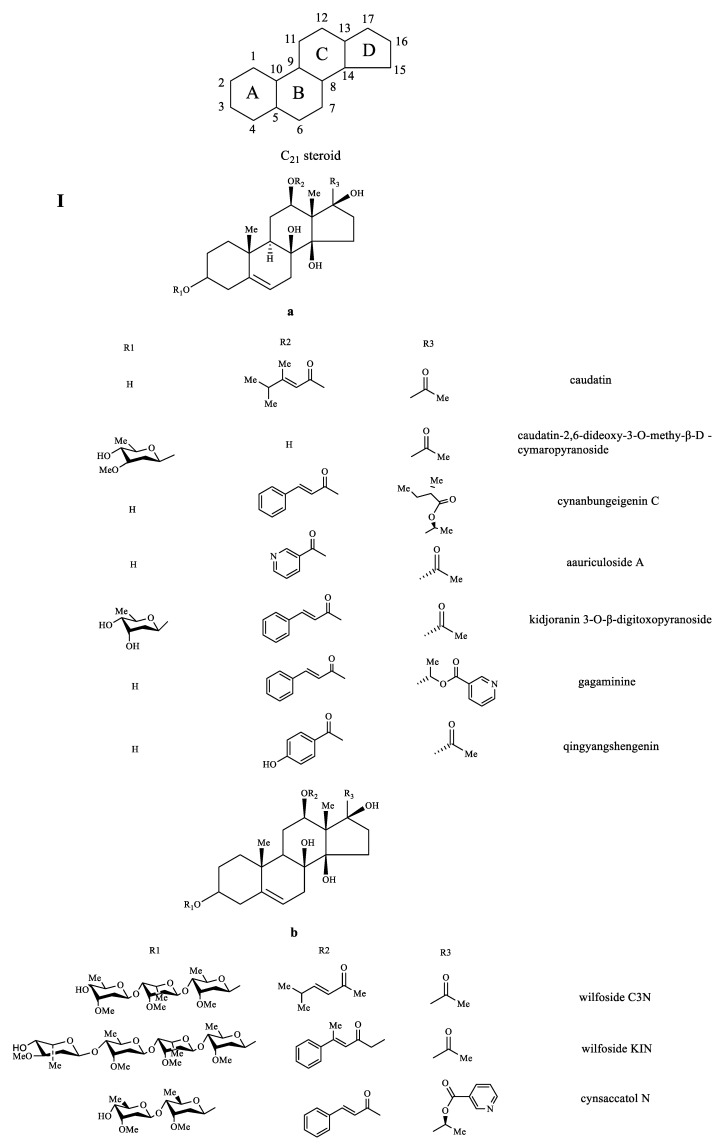

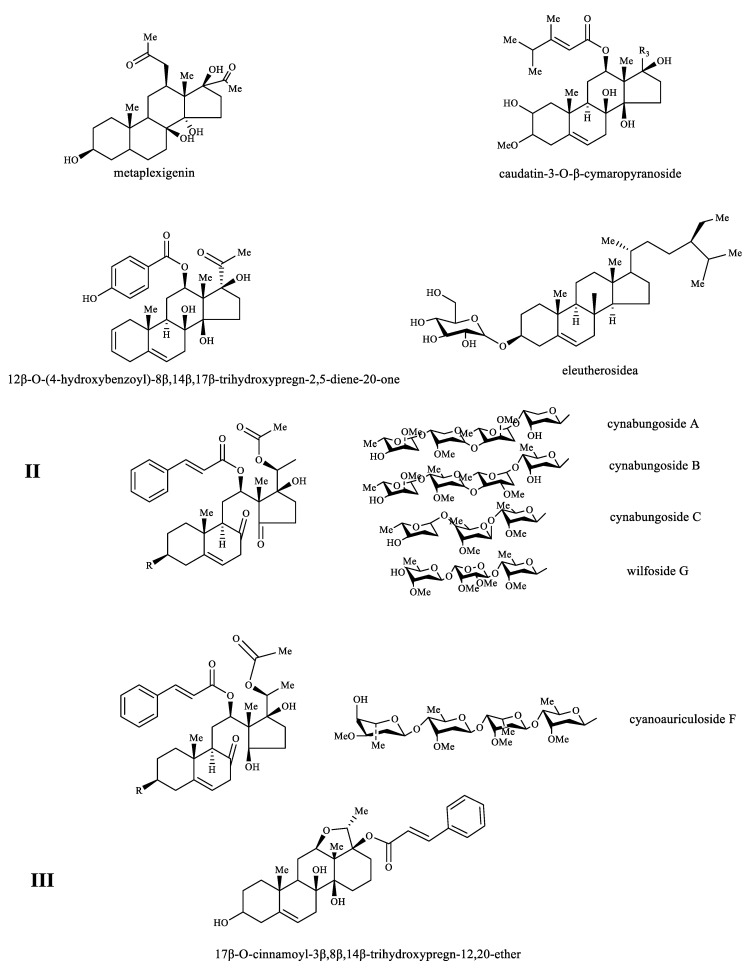
Representative structures of C_21_-steroid compounds isolated from *CA*, *CB* and *CW*. Pregnane (**I**), seco-pregnane (**II**), trihydroxypregnane (**III**).

Previous biological investigations have revealed that caudatin, kidjoranin, qingyangshengenin, gagaminin, and their derivatives exhibited remarkable anti-tumor and antioxidant activities [62,63,64]. Furthermore, the biosynthesis pathway of C_21_-steroids was illustrated by Figure 2 based on relevant references, which was conducive to clarifying their roles in the living organism in the prospective study [65,66,67]. However, the number of bioactive constituents of C_21_-steroids is not much, and further pharmacological study should be carried out. Representative structures of C_21_-steroid compounds isolated from *CA*, *CB,* and *CW* are displayed in Figure 3.

#### 3.2.2. Acetophenones

**Figure 4:**
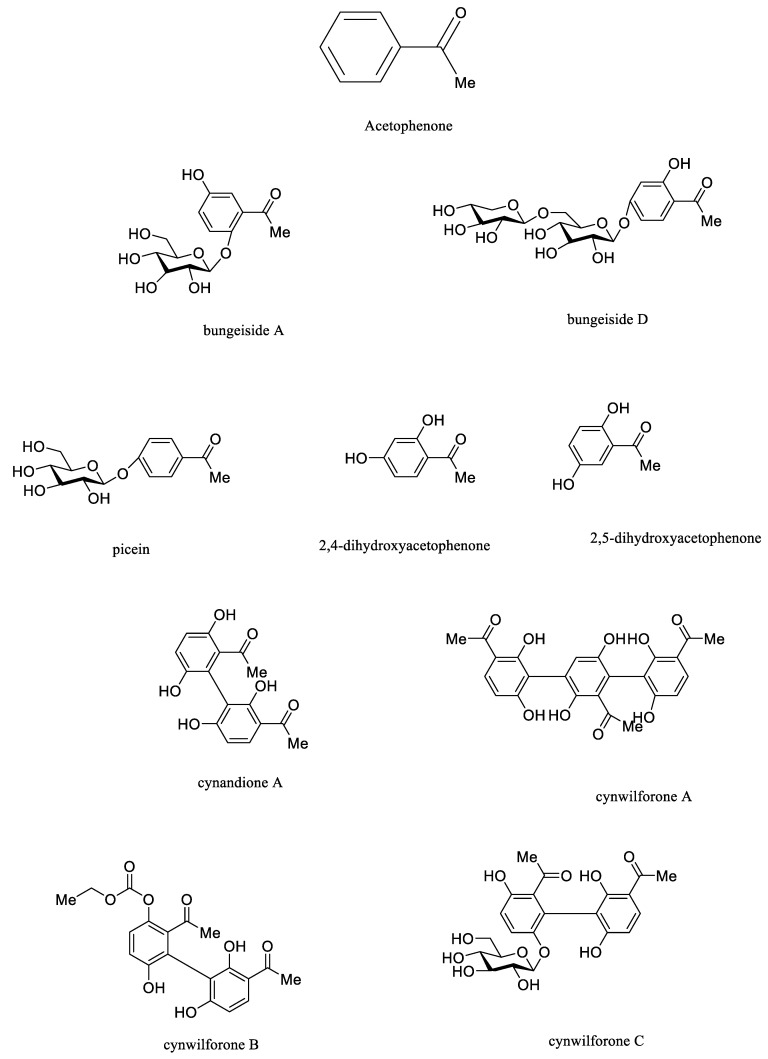
Representative structures of acetophenone compounds isolated from *CA*, *CB* and *CW*.

Acetophenone is the simplest aromatic compound composed of the benzene ring and ketone carbonyl, presenting a colorless or light-yellow liquid, and it is generally found in the volatile oil of plants [68]. Normally, OH groups at C2 and C4 connect with sugar groups among acetophenones isolated from *CA*, *CB*, and *CW*, such as bungeiside A-D and picein. 2,4-dihydroxyacetophenone and 2,5-dihydroxyacetophenones links with biphenyl-like structures, such as cynandione A and cynwilforone A–C. Additionally, cynandione A as the primary bioactive compound in acetophenones has been uncovered to exert obvious neuroprotection and hepatoprotection, reducing liver lipid and blood lipid effects [57,69,70,71]. However, bioactive compounds of acetophenones of *CA*, *CB*, and *CW* were focused on cynandione A, and consequently more compounds with biological properties should be determined in the prospective study. Representative structures of acetophenone compounds isolated from *CA*, *CB* and *CW* were displayed in Figure 4.

#### 3.2.3. Terpenoids and Alkaloids

Terpenoids are important secondary metabolites commonly found in the plant kingdom, and they are olefins with isoprene as the basic unit [72]. According to the number of basic skeletal structures, they can be divided into monoterpenes, sesquiterpenes, diterpenes, and triterpenes. Generally, monoterpenes and sesquiterpenes are volatile oily liquids with a special odor, whereas diterpenes and triterpenes are solid crystals [73]. Notably, sesquiterpenes, such as wilfolides A and wilfolides B, along with triterpenes such as 28α-homo-β-amyrin acetate, cycloartenol, taraxaslero acetate, and betulinic acid, are main presences in three species [13,30,74]. 

**Figure 5:**
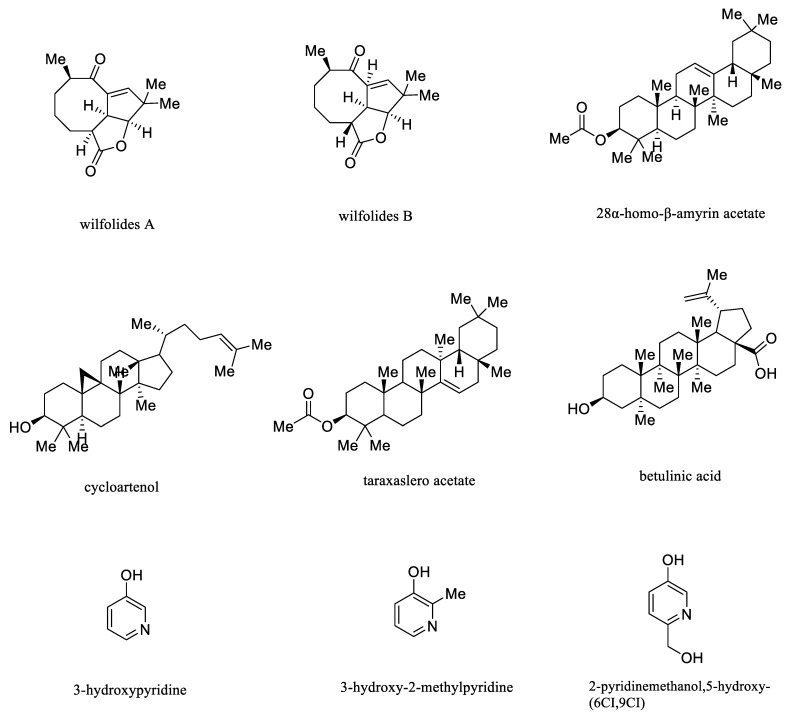
Representative structures of terpenoid and alkaloid compounds isolated from *CA*, *CB* and *CW*.

Alkaloids are nitrogen-containing basic organic substances primarily found in plants, containing nitrogen elements in the ring, exhibiting obvious biological activities in general [75]. Normally, they mainly existed in pyridine type, such as 3-hydroxypyridine, 3-hydroxy-2-methylpyridine, and 2-pyridinemethanol,5-hydroxy-(6CI,9CI) [15]. However, the references regarding the bioactive activity of alkaloids and terpenoids isolated from three species were scarce, requiring further study to unravel their pharmacological effects. Representative structures of terpenoid and alkaloid compounds isolated from *CA*, *CB,* and *CW* were displayed in Figure 5.

#### 3.2.4. Others

Polysaccharide is a polymer carbohydrate composed of more than 10 monosaccharides, which can be divided into homopolysaccharide and heteropolysaccharide based on the constituents of different monosaccharides [76]. It was reported that three heteropolysaccharides with molecular weights of 28,000, 51,000, and 11,700 of *CA* were determined to be composed of rhamnose, arabinose, xylose, mannose, galactose, and glucose [77]. The current study has revealed that polysaccharides of three species exhibited anti-inflammatory, antioxidant, immunomodulatory effects, but their structures have not been determined [78,79,80]. In addition, coumarins, lignans, and phenolic acids were also isolated from *CA*, *CB,* and *CW*, but their pharmacological effects have not been reported in the previous study. Consequently, further study should be conducted to identify exact structures of polysaccharides and explore potential biological activities of other constituents. C_21_-steroids, acetophenones, terpenoids, alkaloids, and other compounds were summarized in Table 1, Table 2, Table 3, Table 4 and Table 5, respectively.

## 4. Pharmacology

### 4.1. Anti-Tumor Activity

To start with, as to hepatic carcinoma, a study revealed that hepatocellular mice induced by diethylnitrosamine model were subjected to the treatment of caudatin of *CA* at the dose of 50 mg/kg, resulting in liver nodule being reduced, and the inflammatory lesion was attenuated obviously, and the further study revealed that restoration of the dysregulated unfolded protein response was responsible for the anti-hepatoma effect of caudatin [25]. In vitro assay, caudatin-2,6-dideoxy-3-*O*-methy-β-d-cymaropyranoside and caudatin isolated from *CA* during the dose of 0.48–300 μM were able to exhibit suppressive effects on SMMC7721 cells with IC_50_ values of 13.49 and 24.95 μM, respectively [92]. C_21_ steroidal glycoside of *CA* within the dose of 45 μg/mL was uncovered to inhibit the proliferation and migration rate of HepG2 cells, leading to apoptotic morphology with nuclear crinkling, hemizygous, or circular shape in a concentration-dependent manner [108]. The ethanol extract of *CA* at the doses of 2 and 4 mg/kg was demonstrated to remarkably inhibit inflammation, liver fibrosis, and hepatocellular carcinoma of mice induced by diethylnitrosamine, and its mechanism was associated with the attenuated expression of TL4 as well as the downregulation of MyD88, TRAF6, NF-κBp65, TGF-β1 and α-SMA [109]. 

For breast cancer, in vivo assay, caudatin of *CA* at the dosage of 10 mg/kg was demonstrated to inhibit proliferation and formation of breast cancer stem cells, and the further study revealed that it induced ubiquitin-dependent glucocorticoid receptor degradation in stem cancer to block Yes-associated protein nuclear accumulation and transcriptional signaling of the target gene [110]. Aauriculoside A, a C_21_ steroidal glycoside isolated from *CA*, was subjected to test MCF-7 cells, resulting in the rate of apoptosis reaching 18.5% at the dose of 40 μg/mL, which was characterized by morphological apoptotic of cytoplasmic contraction and nuclear chromatin condensation [93]. As for uterine cancer, caudatin isolated from *CA* at the concentration ranging from 25 to 100 μmol/L was able to inhibit the proliferation, migration, and induce apoptosis of HeLa cells and endometrial carcinoma cell line [62]. In addition, kidjoranin 3-*O*-α-diginopyranosyl-(1→4)-β-cymaropyranoside and kidjoranin 3-*O*-β-digitoxopyranoside, caudatin 3-*O*-β-cymaropyranoside isolated from *CA* at the concentrations of 10 mM were revealed to exert significant cytotoxic effects on HeLa, SMMC-7721, and MCF-7 cells with IC_50_ values ranging from 8.6 to 58.5 μM, suggesting they have extensive suppressive effects on cervical cancer, hepatoma, and breast cancer [64].

Concerning human glioma, In vitro assay, caudatin isolated from *CB* at the dose of 100 μmol/L enabled the activity of human glioma U251 cells to reach 28.2% in a time-dose dependent manner with IC_50_ value of 52.1 μM [111]. Cynanbungeigin C and cynanbungeigin D isolated from *CB* at the dosage of 50 mg/kg were revealed to block Hedgehog pathway-dependent medulloblastoma by regulating the level of Gli, suggesting they were potential to be developed into new therapeutic agents of human glioma and malignancies [91].

For gastric cancer, the growth of AGS and HGC-27 cells was effectively inhibited with the treatment of caudatin of *CB* during the dose of 25–100 μM in a time-dose dependent manner, and their IC_50_ values were 54.92 and 65.98 μmol/L, respectively [112]. Caudatin 3-*O*-β-d-cymaropyranosyl-(1→4)-β-d-oleandropyranosyl-(1→4)-β-d-cymaropyranosyl-(1→4)-β-d-cymaropyranoside isolated from *CA* within the dose of 80 μM enabled the inhibition rate of HGC-27 cells to reach 93.3% in time-dose dependent manner after 72 h, and its IC_50_ value was 15 μM [113]. Additionally, a study reported that wilfoside C3N of *CA* within the concentration of 16 mg/mL was subjected to treat esophageal cancer ECA109 cells In vitro assay, resulting in 21.3% apoptosis in a time-dose dependent manner [114]. Another study revealed that caudatin of *CB* within the dosage of 100 μg/mL obviously attenuated proliferation and migration of alveolar basal epithelial cell line A549, and eventually inhibited growth and angiogenesis of alveolar basal epithelial cells [115]. 

### 4.2. Neuroprotection and Organ Protection

Intriguingly, neuroprotection, hepatoprotection, gastric protection, and skin protection activities have been uncovered from three species in the previous study, which were characterized by healthcare effects. 

For neuroprotection, it was reported that cynandione A isolated from *CA* at the dosage of 30 mg/kg was able to reduce the area of cerebral infarction of mice by 7.2% in a dose-dependent manner after 72 h, which was correlated with the activity to attenuate glutamate-induced cytotoxicity [116]. Cynsaccatol Q and saccatol K of *CA* during the concentrations of 0.1–10 μM were subjected to treat PC12 cells induced by H_2_O_2_, and as a result, they played a neuroprotective role against oxidative damage by decreasing intracellular ROS and Ca^2+^ levels and inhibiting cell apoptosis [90]. A study reported that (+) cynwiforone F and (−) cynwiforone F of *CW* at the doses of 10 μM possessed a protective effect on SH-SY5Y cells treated with Aβ oligomer, revealing they were capable of increasing cell survival to 76.34% and 81.65%, respectively [100]. Four C_21_ steroidal glycosides, cynsaccatols I, N, O, S isolated from *CA* at the dosages of 1 μM possessed suppressive effects on PC12 cells apoptosis by Annexin V-FITC/PI double staining assay with flow cytometry [97]. 

With respect to hepatoprotection, in hepatocytes in a mice model, induced by CCl_4_, cynandione A isolated from *CW* at the dose of 50 μM, were damaged by regulating the levels of glutathione, superoxide dismutase, catalase, as well as glutathione reductase essential to combat against oxidative stress in cellular defense [69]. C_21_ steroidal glycoside of *CA* at the doses of 4 and 8 g/kg were able to decrease the levels of AST and ALT, improve SOD activity, and reduce malondialdehyde content in the in vivo assay, which significantly inhibited hepatocyte edema and degeneration induced by CCl_4_ [117]. Moreover, polysaccharide of *CA* at the dosage ranging from100 to 500 mg/kg was capable of reducing serum ALT and AST levels in mice with hepatic injury induced by CCl_4_, revealing its feasibility to exert hepatoprotection effect [118]. 

For gastric protection, a study reported that ethanol extracts of *CA*, *CB*, and *CW* at the dosages of 150 and 68 mg/kg possessed potent protective effects on gastric injury induced by ethanol and indomethacin, as well as gastric acid secretion induced by histamine in rats [119]. Another study revealed that water extracts of *CW* at the dosage of 0.72 g/kg enabled serum gastric motility, gastrin, gastric emptying rate, and small intestinal propulsion rate to increase, as well as vasoactive intestinal peptide levels to decrease, which promoted gastric empty and small intestinal propulsion [120]. Additionally, 2,5-dihydroxyacetophenone isolated from *CB* at the dose of 0.4 mM was able to inhibit melanin synthesis and tyrosinase activity of mice melanoma cells stimulated by 3-isobutyl-1-methylxanthine and increase the mean skin lightening index, indicating its potential use as a therapeutic human skin protector [121].

### 4.3. Immunoregulation Activity

It was reported that cyclosporine A as a positive control, nine C_21_-steroid compounds including cynabungosides A-C, wilfoside K1N, wilfoside C1N, 12-*O*-nicotinoylsarcostin-3-*O*-β-lcymaropyranosyl-(1→4)-β-d-cymaropyranosyl-(1→4)-α-l-diginopyranosyl-(1→4)-β-d-cymaropyranoside, deacylmetaplexigenin 3-*O*-α-cymaropyranosyl-(1→4)-β-cymaropyranosyl-(1→4)-α-cymaropyranosyl-(1→4)-β-cymaropyranosyl-(1→4)-β-cymaropyranoside, cynabungone, and cynabungolide within the doses of 40 μM were demonstrated to have potent suppressive effects on T lymphocytes proliferation with IC_50_ values ranging from 1.63 to 40.93 μM [86]. Notably, as to the above compounds except for cynabungone, other eight compounds were uncovered to significantly inhibit the proliferation of B lymphocytes, with IC_50_ values ranging from 0.64 to 38.80 μM [86]. Moreover, a study reported that the crude polysaccharide of *CA* at the doses of 100 and 200 mg/kg was able to improve the expression of nitric oxide, and immunostimulatory cytokines such as interleukin-6 and TNF-α in RAW264.7 macrophages activated by recombinant interferon-γ prime [78]. Meanwhile, it could also recover body weight, immune organ weight, natural killer cell activity, as well as the proliferation of T and B lymphocytes of mice immunosuppressed by cyclophosphamide in vivo assay [78].

### 4.4. Reducing Liver Lipid and Blood Lipid

Cynandione A isolated from *CW* at the dose of 100 μM was revealed to promote the differentiation of adipocyte 3T3-L1 cells by enhancing the expression of lipogenic transcription factors, brown adipocyte-associated genes, and beige adipocyte-associated genes [70]. In a mice model with nonalcoholic fatty liver disease, the ethanol extract of *CW* at the doses of 100 and 200 mg/kg was revealed to reduce hepatic fat accumulation and hepatosplenomegaly damage, and its mechanism was related to the suppressive effects on COX-2, NF-κB, and p38 MAPK [122].

A study reported that ethanol extract of *CW* at the dose of 0.2 g/kg was capable of increasing HDL-cholesterol level and reducing atherosclerotic index of mice with hypercholesterolemia disease, possessing its activity of reducing blood lipid to some extent [123]. Another study uncovered that 2,5-dihydroxyacetophenone and cynandione A isolated from *CW* during the concentrations of 10–40 μM were able to alleviate atherosclerosis by inhibiting LDL oxidation and glycosylation [71]. 

### 4.5. Anti-Inflammatory Activity

Cynandione A of *CW* within the dose of 200 μM was able to significantly decrease the levels of pro-inflammatory cytokines such as TNF-α, IL-6, and IL-1β in mice with LPS, and the further study demonstrated that its mechanism was associated with suppressive effects on NF-κB and MAPK signaling pathways [124]. It was reported that 4-hydroxyacetophenone, cynandione A, and ethanol extract of *CW* within the concentration of 200 μg/mL were found to have a significant suppressive effect on human aortic smooth muscle cells stimulated by TNF-α, illustrating they could be used for the treatment of vascular inflammatory diseases [125]. The crude polysaccharide of *CW* at 100 and 200 mg/kg was demonstrated to improve the pathological features and reduce the production of serum pro-inflammatory cytokines in mice induced by colitis, as well as inhibit several cytokines and enzymes correlated with inflammation by attenuating NF-κB and protein kinase activated by mitogen in RAW264.7 macrophages [79]. C_21_ steroidal glycoside of *CA* within the dose of 45 μg/mL could protect against oxidative toxicity and inflammatory damage in L02 cells induced by H_2_O_2_ through upregulating the expression of Nrf2 and HO-1 via the NF-κB signaling pathway [126]. 

### 4.6. Antioxidant Activity

A study uncovered that the polysaccharide of *CA* within the dose of 12.5 μg/mL exhibited scavenging activity against ABTS, DPPH, and superoxide anion radical in vitro antioxidant models, with IC_50_ values of 0.1232, 0.5543, and 0.5881 mg/mL, respectively [80]. Moreover, it was revealed to increase the content of antioxidant enzyme SOD and non-enzymatic antioxidant GSH in oxidatively damaged cells, which contributed to reducing oxidative stress and achieving intracellular antioxidant effects [61]. Gagaminine, a C_21_-steroidal compound isolated from *CW*, was revealed to have potent suppressive effects on aldehyde oxidase activity and lipid peroxidation at the dose of 2 mg/L in vitro assay [63]. Cynandione A, cynandione B, cynandione E, cynanchone A, and cynantetrone isolated from *CA* at the doses of 1 μM were revealed to exert potent antioxidant activity through inhibiting oxidative damage induced by H_2_O_2_ [105].

### 4.7. Antidepressant Activity

It was reported that a methanol extract of *CA* at the dose of 10 mg/L possessed a 71.1% inhibition rate with an IC_50_ value of 5.2 mg/L, revealing its promising antidepressant role in therapy agents [127]. A study regarded fluoxetine at the dose of 20 mg/kg as the positive control and revealed cynanauriculosides C-E, cynauricuoside C, and otophylloside L isolated from *CA* were able to exhibit potent antidepressant activity at the doses of 50 mg/kg in vivo assay, of which cynanauriculosides D was close to the fluoxetine [27]. 

### 4.8. Antifungal and Antiviral Activities

A study reported that caudatin and qingyangshen of *CW* as leading compounds were applied to synthesize four derivates of C_21_-steroids, including 3-*O*-(methanesulfonoyl)caudatin, 3-*O*-(nicotinic)caudatin, 3,17-*O*-Di(4-methoxycinnamoyl)qingyangshengenin, 3,17-*O*-Di(p-anisoyl)qingyangshengenin, and they were revealed to exhibit significant suppressive effects on the growth of sclerotinia sclerotiorum at the dosages of 50 μg/mL by mycelial growth rate assay, with IC_50_ values of 0.0084, 0.0049, 0.0053, and 0.0034 μM, respectively [128]. It was reported that wilfoside C1N, wilfoside C1G, and wilfoside C1GG isolated from *CW* at the doses of 63 μg/mL were able to possess potent suppressive effects on the activity of *Blumeria graminis* f. sp. Hordei, suggesting they might be used as prominent fungicides to control powdery mildew [26]. A study on *CW* found that its ethyl acetate fraction of ethanol extract at the dose of 40 g/mL possessed potent antiviral activity against the influenza virus with an IC_50_ value of 27.84 μg/mL, uncovering its potential antiviral application in the future [129].

### 4.9. Others

**Figure 6:**
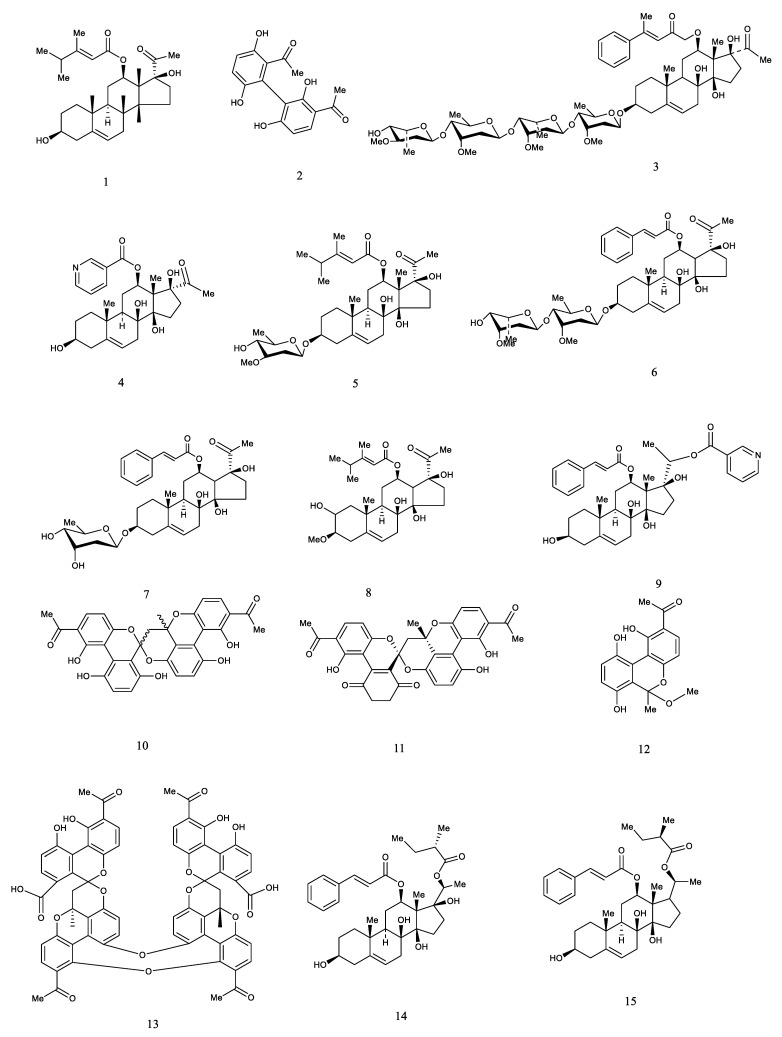

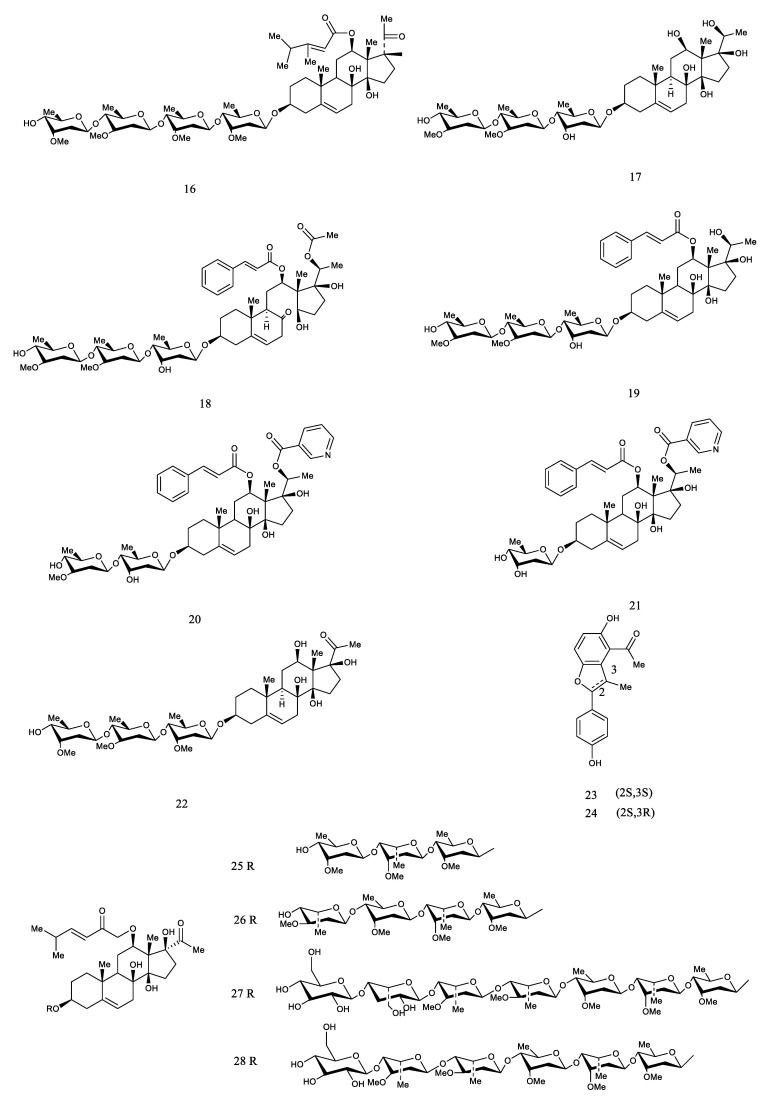

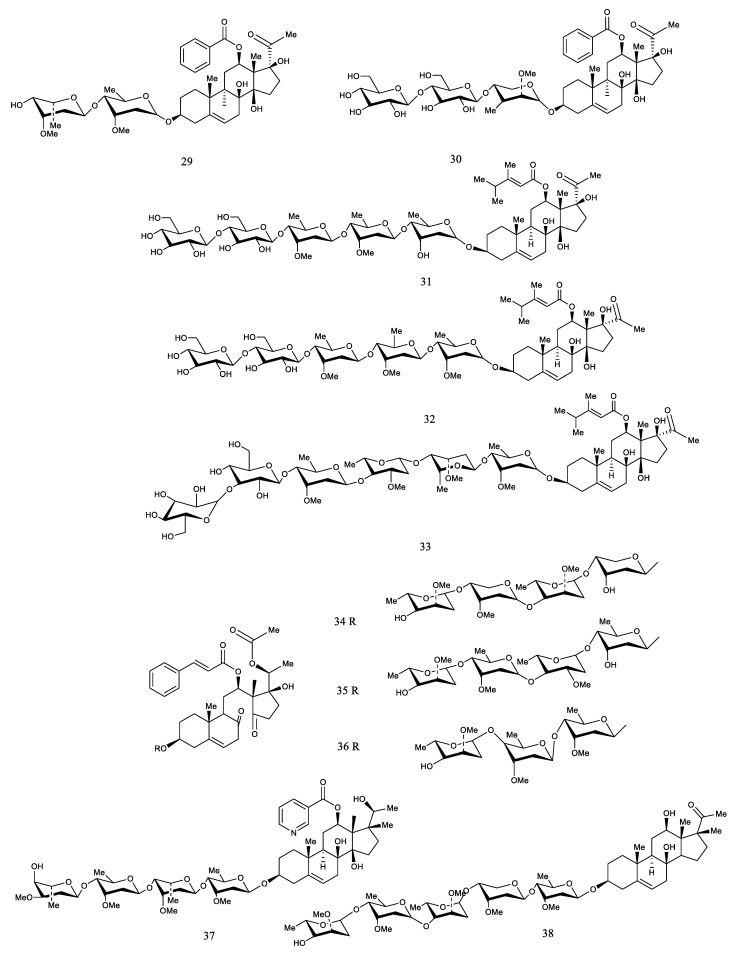

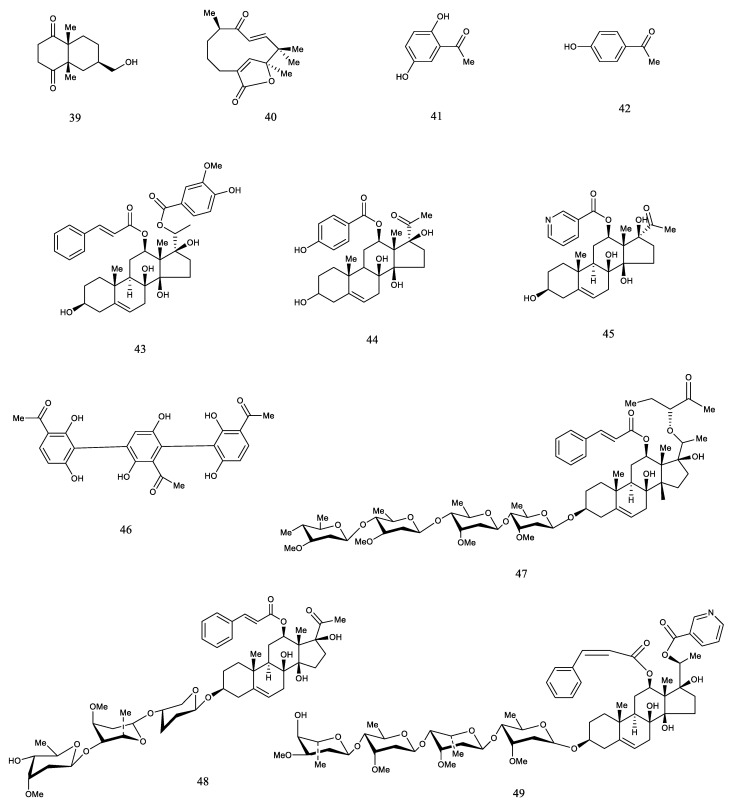
Chemical structures of bioactive compounds isolated from *CA*, *CB* and *CW*.

In addition to the above pharmacological effects, hypoglycemic, anti-angiogenic, anti-prostatic, anti-leukemic, anti-epileptic, appetite suppression, aphrodisiac, menopause suppression, anti-prostatic hyperplasia, and bone-strengthening effects have been uncovered from *CA*, *CB*, and *CW*. A study reported that cynandione A and cynwilforone A isolated from *CW* at the dosages of 40 μM enabled the suppressive rates of hepatic gluconeogenesis to reach 29.2% and 29.4%, respectively, and their mechanism were associated with the downregulation of PEPCK and G6P expressions [99]. Wilfoside KIN of *CW* at the dose of 10 μM displayed suppressive effects on the micro-vessel formation and tube formation of human umbilical vein endothelial cells, suggesting it was potential to be developed into a new anti-angiogenic agent [130]. A study on *CW* revealed that 20-*O*-salicyl-kidjoranin was able to have a significant cytotoxic effect on leukemia cells HL-60 with an IC_50_ value of 6.72 μM, while qingyangshengenin and rostratamin possessed cytotoxic effects on leukemia cells K-562 and MCF-7, and their IC_50_ values were 6.72 and 2.49 μM, respectively [32]. Cynawilfoside A, cynauricoside A, wilfoside C1N, wilfoside K1N, and cyanoauriculoside G isolated from *CW* at the doses of 100 mg/kg possessed anti-epileptic effects on mice induced by maximal electroshock with ED_50_ values of 48.5, 95.3, 124.1, 72.3, and 88.1 mg/kg, respectively [74]. Moreover, wilfoside C1N and wilfoside K1N isolated from *CA* at the doses of 50 mg/kg were able to decrease food consumption, water consumption, and weight of rats, revealing their potent anti-appetite effect [37]. Water extract of *CW* at the dose of 200 mg/kg was employed to improve motivation and libido of male Sprague Dawley rats to exert an aphrodisiac effect through stimulating the secretion of luteinizing hormone, follicle-stimulating hormone, and testosterone [131]. In ovariectomized mice, the water extract of *CW* at the concentration of 40 μg/mL was able to attenuate uterine atrophy and bone loss without changing the plasma estradiol concentration, as well as reduce plasma follicle-stimulating hormone, alkaline phosphatase, and osteocalcin to normal levels, revealing its feasibility to be developed into therapeutic agents for the prevention of menopausal syndrome in women [132]. In mice models with benign prostatic hyperplasia induced by testosterone, water extract of *CW* at the dose of 50 mg/kg could make prostate growth inhibition rate reach 54.5%, which was attributed to the suppressive effects on the expressions of androgen receptor, 5α-reductase, and B-cell lymphoma-2 [133]. Moreover, water extract of *CW* at the dose of 400 mg/kg was capable of enhancing the bone mineral density of mice with osteoclast differentiation and osteoporosis, accompanied by the decreased phosphatase, osteocalcin, and collagen type I C-telopeptide, and tartrate-resistant acid phosphatase levels [134]. Subsequently, it was also revealed to reduce bone marrow cells as well as increase bone mineral density and profile, suggesting it possessed a potent therapeutic effect on osteoporosis [135]. Chemical structures of bioactive compounds isolated from *CA*, *CB*, and *CW* are displayed in Figure 6. The pharmacological effects of bioactive compounds and extracts isolated from *CA*, *CB,* and *CW* are shown in Table 6.

## 5. Molecular Mechanism

Since phytochemicals are usually characterized by multi-pathways and multi-targets in the living organism, it is difficult for us to clarify their mechanisms comprehensively and clearly. Given that, we screened the representative molecular pathways and action factors of bioactive compounds and extracts in *CA*, *CB,* and *CW* to provide a convenient understanding for readers.

### 5.1. Cell Cycle Arrest

The cell cycle can be divided into G0, G1, S, G2, and M phases, and some bioactive compounds can inhibit their intracellular genomic DNA and block the process of mitosis to cause the suppressive effect on cell proliferation [138]. A study revealed that alveolar epithelial cell line A549 was blocked at the G0/G1 phase in a dose-dependent manner with the treatment of caudatin of *CB* within the concentration of 100 µg/mL, and its IC_50_ value was 121.1 mg/mL [115]. Gastric cancer cells AGS and HGC-27 were treated by caudatin of *CB* during the dose of 25–100 µM, and revealed that G1 to S phase metastasis was blocked in a dose-dependent manner through downregulating CDK2 protein levels [139]. Hepatocellular carcinoma SMMC-7721 cells were tested by caudatin of *CA* at the dose of 12.5µM, leading to the number of G2 cells having a significant increase than S phase in a time-dose dependent manner, which was attributed to blocking the transportation of the S to G2 phase [136]. A study concerning human glioma U251 and U87 cells reported that caudatin of *CB* during the dose of 25–100 µM triggered cell arrests of G0/G1 and S phases, which significantly inhibited the proliferation-related cell to upregulate p53, p2, and histone phosphorylation, as well as downregulated cyclinD1 [140]. When MCF-7 cells were treated by the auriculoside A of *CA* at the dosage of 40 µg/mL, the number of hepatocellular carcinoma MCF-7 cells was increased in G0/G1 phase whereas was decreased in S and G2/M phases, indicating that it enabled cell cycle arrest to locate in G0/G1 phase [93].

### 5.2. Triggering Cell Apoptosis

Cell apoptosis is an autonomous and ordered death to maintain the stability of the internal environment, characterized by cell volume contraction, chromatin condensation, shedding from its surrounding tissues, and phagocytosis with no inflammatory response from the organism [141]. Current research on *CA*, *CB,* and *CW* has revealed that the mechanism of cell apoptosis was associated with the bc-2 family and caspase family. In hepatocellular carcinoma HepG2 cells with the treatment of caudatin of *CB* during the dose of 25–100 μM, the expression of Bcl-2 was downregulated, whereas Bax expression was upregulated, accompanied by activation of caspase-3, -9, and polymerase [139]. C_21_-steroidal glycoside of *CA* at the dosage of 21.6 μM was subjected to treat gastric cancer SGC-7901 cells to cause a 43.2% apoptosis rate, and the further study revealed that it could trigger apoptosis through the caspase-3 dependent pathway [113]. A report uncovered that human glioma U251 and U87 cells were triggered with the treatment of caudatin during the dose of 25–100 μM in caspase-dependent apoptosis through mitochondrial dysfunction and reactive oxygen species production [111]. Additionally, wilfoside C3N isolated from *CA* during the dose of 2–32 μg/mL was able to trigger apoptosis of esophageal cancer ECA109 cells through triggering the release of cytochromes from mitochondria and activating caspase-9 receptors [114].

### 5.3. Effect on NF-κB Pathway

NF-κB is an important intracellular nuclear transcription factor that is mainly involved in anti-inflammatory, anti-tumor, and immunomodulatory effects in humans [142]. IκB including common IκBα and IκBβ, as an inhibitory protein of NF-κB, is normally combined with NF-κB to prevent NF-κB translocating into the nucleus when it was activated by bioactive constituents [143]. The suppressive effects on NF-κB contributed to enhancing the expression of IL-6, TNF-α, and IL-1β cytokines to treat inflammatory disease. Of note, current investigations regarding *CA*, *CB,* and *CW* have demonstrated that the NF-κB pathway was primarily correlated with anti-inflammatory effects. For example, cynandione A of *CW* within the dose of 50 μM was uncovered to possess a potent suppressive effect on BV-2 microglial cells induced by LPS, and its mechanism was demonstrated to be related to the suppressive effect on the phosphorylation of IκBα and the translocation of nuclear factor NF-κB to BV-2 cells [144]. A study revealed that caudatin of *CA* at the concentration of 100 µmol/L was able to inhibit the proliferation and migration of human cervical carcinoma cell lines and endometrial carcinoma cell lines through the TNFAIP1/NF-κB signaling pathway [62]. Another study on hepatocyte cell lines L02 induced by H_2_O_2_ revealed that C_21_-steroidal glycoside of *CA* within the dose of 45 μg/mL was able to protect them from oxidative toxicity and inflammatory damages by enhancing Nrf2 and HO-1 expression via the NF-κB signaling pathway [126]. 

### 5.4. Effect on MAPK Pathway

MAPK containing ERK, p38, JNK, and ERK5 subfamilies is an important transmitter from the cell surface to the interior of the nucleus, and it can be expressed in all eukaryotic cells involving in cell proliferation, differentiation, apoptosis, inflammation, and other activities [145]. In mice models with neurological disease, cynandione A of *CW* at the dose of 100 μM was able to upregulate the phosphorylation of MAPK including p38, ERK1/2, and JNK, thereby leading to promote β-endorphin expression and reduce neuropathic pathological pain [146]. In an investigation regarding TRAIL-induced apoptosis in human breast cancer cells, caudatin of *CA* within the concentration of 100 μg/mL contributed to promoting DR receptor expression to trigger apoptosis by increasing CHOP expression and phosphorylation of p38MAPK and JNK [147]. In mice fed with a high-fat and high-fructose diet, ethanol extract of *CW* at the dosages of 100 and 200 mg/kg could reduce fat accumulation and damage in the liver by inhibiting p38MAPK and AKT phosphorylation [122]. Primary human skin fibroblast and human keratinocyte models were established to uncover that polysaccharides of *CW* at the doses of 200 and 400 μg/mL could significantly suppress UVB-induced oxidative stress, connected with the mechanisms to downregulate MKK4-JNK, MEK-ERK, and MKK3/6-p38 phosphorylation [148].

### 5.5. Effect on Wnt/*β*-Catenin Pathway

The wnt/β-catenin pathway is commonly associated with cancer diseases, of which wnt is a secreted glycoprotein that leads to the accumulation of β-catenin, while β-catenin is a bifunctional protein that regulates cell-cell adhesion and the coordination of gene transcription to promote cell proliferation [149]. GSK3β is a key member of the wnt signaling pathway, and it often combines with β-catenin when wnt protein disappears. A study revealed that caudatin of *CB* within the concentration of 100 μg/mL could inhibit the growth of human alveolar carcinoma basal epithelial cells through the GSK3β/β-catenin pathway [115]. In gastric cancer HGC-27 cells, caudatin of *CB* at the dose ranging from 25 to 100 μM was able to decrease β-catenin expression that was caused by the downregulation of cyclinD1 and c-myc, suggesting wnt/β-catenin signaling pathway was correlated with the treatment of caudatin in gastric cancer [112]. Additionally, caudatin of *CW* during the dose of 12.5–50 μM was uncovered to inhibit GSK3β and β-catenin expression, which was attributed to the suppressive effect on wnt protein target genes COX-2, MMP2, and MMP9 [136].

### 5.6. Effect on Vascular Factor

**Figure 7:**
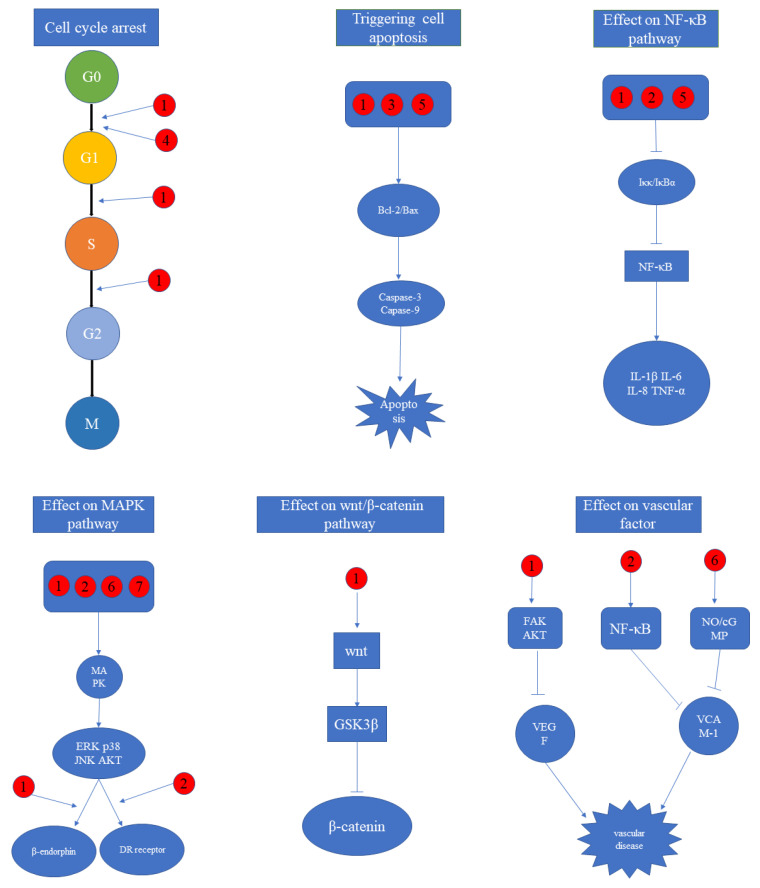
Representative molecular mechanism of bioactive compounds and extracts isolated from *CA*, *CB* and *CW.* (Bioactive constituents: ①: Caudatin, ②: Cynandione A, ③: Wilfoside C3N, ④: Auriculoside A, ⑤: C_21_ steroidal glycoside of *CA*, ⑥: Ethanol extract of *CW*, ⑦: Polysaccharide of *CW*).

Current research regarding *CA*, *CB* and *CW* revealed that developing therapeutic agents based on anti-angiogenesis and vascular protection were primary directions for the treatment of vascular disease. Anti-angiogenic therapy is a novel and effective method to treat the disease that was dependent on angiogenesis, while vascular protection was attributed to suppressive effects on VEGF and VCAM factors [150]. An anti-angiogenic study reported that caudatin of *CA* during the dose of 50–200 μM was able to inhibit proliferation, migration, invasion, and vascular formation of human umbilical vein endothelial cells by interfering with the vascular endothelial growth pathway of VEGF-VEGFR2-AKT/FAK [151]. A study regarding vascular protection revealed that cynandione A of *CW* within the dose of 40 μM could effectively inhibit VCAM-1 expression in LPS-induced umbilical vein endothelial cells and human aortic smooth muscle cells stimulated by TNF-α [137]. Furthermore, in mice models fed with a high-fat/cholesterol diet, ethanol extract of *CW* at the dosage of 200 mg/kg was revealed to ameliorate hypertension and endothelial dysfunction through NO/cGMP signaling pathway, associated with the suppressive effect on ET-1, VCAM, and lesion formation, which could be applied to the treatment and prevention of atherosclerotic vascular disease [152]. Representative molecular mechanisms of bioactive compounds and extracts isolated from *CA*, *CB,* and *CW* were displayed in Figure 7.

## 6. Pharmacokinetics

Pharmacokinetics is used to evaluate and explore quantification, absorption, distribution, and metabolism patterns of bioactive constituents in living organisms, which is an important criterion for evaluating potential new drugs [153]. However, bioactive constituents regarding pharmacokinetic of *CA*, *CB*, and *CW* are not enough and they are mainly concentrated on cynandione A, caudatin and its derivatives. It was reported that cynandione A of *CW* at the dosage of 2.5 mg/kg was employed to evaluate by LC-MS/MS method in vivo assay, resulting in retention time of 1.41 min and concentration range of 0.2–1000 ng/mL, good linearity with intra-day and inter-day, and precision and accuracy of NE in plasma and tissues less than 14.4%, suggesting it could be absorbed and distributed rapidly without long-term accumulation in mice tissue [154]. The determination of caudatin of *CB* at the dosage of 12 mg/kg was established in rats plasma by UPLC-MS/MS using carbamazepine as an internal standard, leading to good linearity in the concentration ranging from 2.5 to 300 ng/mL, as well as precision and accuracy of inter-day and intra-day were within 10% and 5%, respectively [155]. In mice with hepatoma induced by diethylnitrosamine, caudatin-2,6-dideoxy-3-*O*-methy-β-d-cymaropyranoside of *CA* during the concentration of 0.1–10 μg/mL was subjected to test by UPLC-MS/MS method, resulting in a linear ranging from 5 to 500 ng/mL, and precision of inter-day and intra-day were within 8.5% and 9.1%, respectively, along with ±10.9% accuracy [156]. Another study determined metabolites of wilfoside C1N and wilfoside C3N of *CB* at the doses of 50 mg/kg in mice plasma and tumor homogenate by the analysis of LC-MS/MS, leading to wilfoside C3N having a better absorption than wilfoside C1N, which might be attributed to different lengths of sugar chain [157].

## 7. Clinical Safety

In 2015, a woman developed eosinophilic esophagitis after 2–3 weeks of repeated *CW* administration, which attracted widespread attention about the safety of *CW*. However, *CW* as a new dietary supplement has been approved by the US Food and Drug Administration for many years, and it has never been reported that there was serious side effect in the body after taking food containing *CW*. Unexpectedly, the case that repeated *CW* administration contributed to causing eosinophilic esophagitis has been confirmed by history, endoscopy, histology, and BAT methods [158]. Given that, clinical trials regarding the safety of *CW* administration have been established. In 2019, ethanol extracts of *CW* at the dosages of 300 and 600 mg/d were subjected to test 84 subjects in a randomized placebo-controlled trial, leading to some adverse events such as urinary discomfort, allergic reactions, vomiting, skin problems, and gastrointestinal discomfort [159]. However, all these events were mild reactions and not statistically significant. Subsequently, 64 subjects with high cholesterol were selected to take ethanol extract of *CW* at the dosages of 300 and 600 mg/d in 2020, resulting in four participants with emerging symptoms of blepharospasm, constipation, vomiting, and pruritus [160]. The manifestation of these symptoms was mild enough with low toxicity that no medication was required and there were no significant abnormalities based on the complete blood counts and blood chemistry results. According to the above cases, we found that *CW* possessed significant adverse effects when it was repeatedly administered, while there were no serious effects on the human body in a mild dose, suggesting it should be consumed in certain doses and not in excess. In previous pharmacological assays, bioactive constituents of *CA*, *CB*, and *CW* have not exerted obvious toxicity in vivo and vitro assays but lacked professional research on their toxicity. To our knowledge, there was no report regarding the clinical trial of *CA* and *CB* in the previous study. Consequently, it is necessary for us to establish clinical trials of *CA* and *CB* to test their safety, which contributed to developing the potential drugs in the future.

## 8. Conclusions and Prospects

First, botany study based on chemical analysis and molecular techniques revealed that there were close relationships among *CA*, *CB*, and *CW*. At the same time, they could also be identified by chemotaxonomic and molecular markers. However, there was a lack of molecular markers of *CA* and *CW*, and therefore molecular techniques of *CB* should be carried out in the future. Second, phytochemistry clarified 232 chemical compounds isolated from *CA*, *CB,* and *CW*, including C_21_ steroids, acetophenones, terpenoids, alkaloids, and others. Nevertheless, they were all only isolated from the roots of three species, and accordingly, more compounds should be found from their stems, leaves, and flowers. Third, pharmacology study uncovered that anti-tumor, neuroprotection, organ protection, reducing blood lipid and liver lipid, immunomodulatory, anti-inflammatory, anti-oxidant, anti-depressant, anti-fungal, and anti-viral effects in three species. Of note, relevant bioactive compounds are not many, emphasizing caudatin and cynandione A, and consequently more bioactive compounds should be uncovered by plenty of pharmacological experiments. Furthermore, concrete compounds of crude extracts with biological activities need to be further determined, which was conducive to clarifying their roles in the further pharmacological assay. Fourth, molecular mechanism study illustrated that cell cycle arrest, triggering cell apoptosis and wnt/β-catenin pathway are primary mechanisms of anti-tumor activity, while NF-κB pathway was responsible for anti-inflammatory activity. MAPK pathway and the role of vascular factor were representative mechanisms to exert neuroprotection and organ protection activities. Undoubtedly, the molecular mechanisms of bioactive compounds isolated from three species are mainly concentrated on typical pathways and targets, and consequently deeper study should be required to uncover new action pathways and targets, as well as explore their feasibilities to develop into new drugs. Fifth, pharmacokinetics study demonstrated that cynandione A, caudatin and its derivatives, were able to exert good absorption, distribution, and metabolism in the living organism. Sixth, clinical trials uncovered that there were no significant adverse effects in the mild dose of *CW*, but required us to strictly control the dose taken. Unfortunately, clinical trials on *CA* and *CB* have not been established, and the ethanol extract was not suitable for developing into new drugs. In the future, bioactive compounds of crude extract should be further confirmed, as well as more clinical trials on *CA* and *CB* should be also carried out. 

Although there were no specialized clinical trials to prove the safety of caudatin and cynandione A, they were mainly obtained in ethanol extract and no relevant toxicity was reported in previous pharmacological experiments, which probably indicated that they have low toxicity to some extent, but the perspective needed to be proved by specialized toxicity tests in the prospective study. According to their pharmacological effects, molecular mechanism, pharmacokinetics, and clinical safety investigations, caudatin was potential to be developed into a new anti-tumor candidate, while cynandione A with extensive organ protection and neuroprotection activities might be made into a promising healthcare agent, but more evidence should be found to support that in the future. In this paper, the current research status of *CA*, *CB*, and *CW* was reviewed, which might be relevant for researchers to acquire a deeper understanding.

## Figures and Tables

**Table 1:** C_21_-steroids glycosides compounds isolated from *CA*, *CB* and *CW*.

Number	Compounds	Species	References
1	Caudatin	*CA CB CW*	[25,26,27]
2	Qingyangshengenin	*CA*	[31]
3	Deacylmetaplexigenin	*CA CW*	[31,32]
4	Rostratamine	*CA CW*	[31,32]
5	Gagaminine	*CA*	[31]
6	Daucosterol	*CA*	[31]
7	Cautatin-3-*O-*β-d-digitoxo-pyranoside	*CA*	[31]
8	Caudatin-3-*O-*β-d-cymaropyranosyl-(1→4)-β-d-digitoxopyranoside	*CA*	[31]
9	Caudatin 3-*O-*β-d-cymaropyranosyl-(1→4)-β-d-cymaropyranoside	*CA*	[31]
10	Otophylloside B	*CA*	[31]
11	Caudatin 3-*O-*β-d-oleandropyranosyl-(1→4)-β-d-digitoxopyranosyl-(1→4)-β-d-cymaropyranoside	*CA*	[31]
12	Gagaminine3-*O-*α-l-cymropyranosyl-(1→4)-β-d-cymropyranosyl-(1→4)-β-d-cymropyranoside	*CA*	[31]
13	Auriculosides A	*CA*	[81]
14	Auriculosides B	*CA*	[81]
15	Cyanoauriculoside C	*CA*	[82]
16	Cyanoauriculoside D	*CA*	[82]
17	Cyanoauriculoside E	*CA*	[82]
18	Cynanauriculoside II	*CA*	[82]
19	Wilfoside K1N	*CA*	[82]
20	Auriculoside IV	*CA*	[82]
21	Kidjolanin	*CA*	[83]
22	3-Formyloxymetaplexigenin	*CA*	[84]
23	Metaplexigenin	*CA*	[84]
24	Auriculoside I	*CA*	[33]
25	Auriculoside II	*CA*	[33]
26	Auriculoside III	*CA*	[33]
27	Wilfoside C1G	*CA CW*	[33]
28	Cynanauriculoside I	*CA*	[33]
29	Cynauricuoside A	*CA*	[33]
30	Gagaminin3-*O-*β-l-cymaropyranosyl-(1→4)-β*-*d-cymaropyranosyl-(1→4)-α-l-diginopyranosyl-(1→4)*-*β-d-digitoxopyranoside	*CB*	[85]
31	Gagaminin3-*O-*β-L-cymaropyranosyl-(1→4)-β-d-cymaropyranosyl-(1→4)-α-l-diginopyranosyl-(1→4)-β-d-cymaropyranoside	*CB*	[85]
32	12-*O*-nicotinoylsarcostin3-*O-*β-l-cymaropyranosyl-(1→4)-β-d-cymaropyranosyl-(1→4)-α-l-diginopyranosyl-(1→4)-β-d-cymaropyranoside	*CB*	[85]
33	Penupogenin3-*O-*β-d-glucopyranosyl-(1→4)-β-l-cymaropyranosyl-(1→4)-β-d-cymaropyranosyl-(1→4)-α-l-diginopyranosyl-(1→4)-β-d-cymaropyranoside	*CB*	[85]
34	12-*O*-acetylsarcostin3-*O-*β-l-cymaropyranosyl-(1→4)*-*β-d-cymaropyranosyl-(1→4)-β-l-cymaropyranosyl-(1→4)-β-d-digitoxopyranosyl-(1→4)-β-d-digitoxopyranoside	*CB*	[85]
35	12-*O*-acetylsarcostin3-*O-*β-l-cymaropyranosyl-(1→4)-β-d-digitoxopyranosyl-(1→4)-β-l-cymaropyranosyl-(1→4)-β-d-cymaropyranosyl-(1→4)-α-l-diginopyranosy-(1→4)*-*β-d-cymaropyranoside	*CB*	[85]
36	Cynabungoside A	*CB CW*	[26,86]
37	Cynabungoside B	*CB*	[86]
38	Cynabungoside C	*CB*	[86]
39	Cynabungolide	*CB*	[86]
40	Cynabungone	*CB*	[86]
41	Wilfoside C1N	*CB*	[86]
42	Wilfolide A	*CB*	[86]
43	Deacylmetaplexigenin3-*O*-α-cymaropyranosyl-(1→4)-β-cymaropyranosyl-(1→4)-α-cymaropyranosyl-(1→4)-β-cymaropyranosyl-(1→4)-β-cymaropyranoside	*CB*	[86]
44	Cynanbungeinoside A	*CB*	[87]
45	Cynanbungeinoside B	*CB*	[87]
46	Cynanbungeinoside C	*CB*	[87]
47	Cynanbungeinoside D	*CB*	[87]
48	Cynanbungeinoside E	*CB*	[87]
49	Cynanbungeinoside F	*CB*	[87]
50	Wilfoside B	*CW*	[88]
51	Wilfoside C	*CW*	[88]
52	Wilfoside D	*CW*	[88]
53	Wilfoside E	*CW*	[88]
54	Wilfoside F	*CW*	[88]
55	Wilfoside G	*CW*	[88]
56	Wilfoside H	*CW*	[88]
57	20-*O*-salicyl-kidjoranin	*CW*	[32]
58	12β-*O*-(4-hydroxybenzoyl)-8β,14β,17β-trihydroxypregn2,5-diene-20-one	*CW*	[32]
59	3-*O*-methyl-caudatin	*CW*	[32]
60	20-*O*-(4-hydroxybenzoyl)-kidjoranin	*CW*	[32]
61	20-*O*-vanilloyl-kidjoranin	*CW*	[32]
62	12-*O*-vanilloyl-deacymetaplexigenin	*CW*	[32]
63	17β*-O*-cinnamoyl-3β,8β,14β-trihydroxypregn-12,20-ether	*CW*	[32]
64	Deacymetaplexigenin	*CW*	[32]
65	12-*O*-benzoyldeacymetaplexigenin	*CW*	[32]
66	12β*-O*-benzoyl8β,14β,17β-trihydroxypregn-2,5-diene-20-one	*CW*	[32]
67	Sarcostin	*CW*	[32]
68	Deacylcynanchogenin	*CW*	[32]
69	Kidjoranine3-*O-*β-d-glucopyranosyl(1→4)-α-l-cymaropyranosyl(1→4)-β-d-cymaropyranosyl(1→4)-α-l-diginopyranosyl(1→4)-β-d-cymaropyranoside	*CB*	[89]
70	Caudatin3-*O-*β-d-glucopyranosyl(1→4)-α-l-cymaropyranosyl(1→4)-β-d-cymaropyranosyl(1→4)-α-l-diginopyranosyl(1→4)-β-d-cymaropyranoside	*CB*	[89]
71	Kidjoranine3-*O-*β-d-glucopyranosyl(1→4)-α-l-diginopyranosyl(1→4)-β-d-cymaropyranoside	*CB*	[89]
72	*O*-nicotinoyl-20-*O*-cinnamonacylsarcostin-*O-*β-d-glucopyranosyl(1→4)-α-l-cymaropyranosyl(1→4)-β-d-cymaropyranosyl(1→4)-α-l-diginopyranosyl(1→4)-β-d-digitoxopyranoside	*CB*	[89]
73	Penupogenin3-*O*-β-d-glucopyranosyl(1→4)-α-l-cymaropyranosyl(1→4)-β-d-cymaropyranosyl(1→4)-α-l-diginopyranosyl(1→4)-β-d-digitoxopyranoside	*CB*	[89]
74	Kidjoranine3-*O-*β-d-glucopyranosyl(1→4)-α-l-cymaropyranosyl(1→4)-β-d-cymaropyranosyl(1→4)-α*-*l-diginopyranosyl(1→4)*-*β-d-digitoxopyranoside	*CB*	[89]
75	Caudatin3-*O-*β-d-glucopyranosyl(1→4)-α-l-diginopyranosyl(1→4)-β-d-cymaropyranoside	*CB*	[89]
76	12-*O*-benzoyl-deacetylmetaplexigenin3-*O-*β-d-glucopyranosyl(1→4)-α-l-cymaropyranosyl(1→4)-β-d-cymaropyranosyl(1→4)-α-L-diginopyranosyl(1→4)-β-d-cymaropyranosi-de	*CB*	[89]
77	Caudatin3-*O-*β-d-glucopyranosyl(1→4)-α-l-cymaropyranosyl(1→4)*-*β-d-cymaropyranosyl(1→4)-α-l-cymaropyranosyl(1→4)-β-d-cymaropyranosyl(1→4)-α-l-diginopyranosyl(1→4)*-*β-d-cymaropyranoside	*CB*	[89]
78	Caudatin3-*O-*α-l-cymaropyranosyl(1→4)*-*β-d-cymaropyranosyl(1→4)-α-l-diginopyranosyl(1→4)-β-d-cymaropyran-oside	*CB*	[89]
79	Caudatin3-*O-*α-l-diginopyranosyl(1→4)-β-d-cymaropyran-oside	*CB*	[89]
80	Kidjoranine3-*O-*β-d-cymaropyranosyl(1→4)-α-l-diginopyranosyl(1→4)-β-d-cymaropyranoside	*CB*	[89]
81	Kidjoranine3-*O-*α-l-diginopyranosyl(1→4)-β-d-cymaropyranoside	*CB*	[89]
82	Caudatin3-*O-*β-d-cymaropyranosyl(1→4)-α-l-diginopy-ranosyl(1→4)-β-d-cymaropyranoside	*CB*	[89]
83	*O*-benzoyl-deacetylmetaplexigenin	*CB*	[89]
84	12-*O*-acetyl-20-*O*-(2-methylbutanoyl)-sarcostin	*CB*	[89]
85	20-*O*-Acetyl-penupogenin	*CB*	[89]
86	Gracigenin	*CB*	[89]
87	8,14-seco-caudatin	*CB*	[89]
88	Penupogenin	*CB*	[89]
89	(20S)-12-cinnamoyloxy-20-acetyloxy-3,5,17-trihydroxy-8,14-seco-5,17-pregn-6-ene-8,14-dione	*CB*	[89]
90	Isoikemagenin	*CB*	[89]
91	*O*-cinnamoyl-20-*O*-(S)-(+)-α-methylbutyryl–sarcostin	*CB*	[89]
92	12-*O*-cinnamoyl-20-*O*-(R)-(-)-α-methylbutyrylsarcostin	*CB*	[89]
93	Cynanauriculoside C	*CA*	[27]
94	Cynanauriculoside D	*CA*	[27]
95	Cynanauriculoside E	*CA CW*	[27,34]
96	Otophylloside L	*CA*	[27]
97	Wilfoside C3N	*CA*	[35]
98	Kidjoranin	*CA CW*	[35]
99	Cynanauriculoside I	*CA*	[35]
100	Cynanauriculoside II	*CA*	[35]
101	Cyanoauriculoside F	*CA*	[82]
102	Cyanoauriculoside G	*CA*	[82]
103	Cyanoauriculoside H	*CA*	[82]
104	3-*O-*α-l-cymaropyranosyl-(1→4)-β-Dcymaropyranosyl-(1→4)-α-l-diginopyranosyl-(1→4)-β-d-cymaropyranoside	*CA*	[82]
105	Wilfoside D1N	*CA*	[82]
106	Cyanoauriculoside A	*CA CW*	[36]
107	Cyanoauriculoside B	*CA*	[36]
108	Cynsaccatol Q	*CA*	[90]
109	Saccatol K	*CA*	[90]
110	Cynanbungeigenin C	*CB*	[91]
111	Cynanbungeigenin D	*CB*	[91]
112	Wilfoside A	*CW*	[88]
113	Wilfoside C1G	*CA*	[92]
114	Cynauricuoside C	*CA*	[92]
115	Caudatin-2,6-dideoxy-3-*O*-methy-β-d-cymaropyranoside	*CA*	[92]
116	Kidjoranin3-*O*-αdiginopyranosyl-(1→4)-β-cymaropyranosi-de	*CA*	[64]
117	Kidjoranin 3-*O-*β-digitoxopyranoside	*CA*	[64]
118	Caudatin 3-*O-*β-cymaropyranoside	*CA*	[64]
119	Auriculoside A	*CA*	[93]
120	Wilfoside C1GG	*CW*	[26]
121	Wilfoside C1G	*CW*	[26]
122	Wilfoside K1GG	*CW*	[26]
123	Wilfoside M1N	*CA CW*	[34,37]
124	Cynauricuside A	*CW*	[37]
125	Cynauricoside B	*CW*	[37]
126	Cynauricoside C	*CW*	[37]
127	Cynauricoside D	*CW*	[37]
128	Cynauricoside E	*CW*	[37]
129	Cynauricoside F	*CW*	[37]
130	Cynauricoside G	*CW*	[37]
131	Cynauricoside H	*CW*	[37]
132	Cynauricoside I	*CW*	[37]
133	Eleutherosidea	*CA*	[38]
134	Caudatin-3-*O-*β-cymaropyranoside	*CA*	[59]
135	Cynauricuoside B	*CA*	[94]
136	Gagaminin-3-*O-*α-l-cymaropyranosyl-(1→4)-β-d-cymaropyranosyl-(1→4)-α-l-diginopyranosyl-(1→4)-β-d–digitoxopyranoside	*CW*	[74]
137	Cynawilfoside A	*CW*	[74]
138	Cynawilfoside B	*CW*	[74]
139	Cynawilfoside C	*CW*	[74]
140	Cynawilfoside D	*CW*	[74]
141	Cynawilfoside E	*CW*	[74]
142	Cynawilfoside F	*CW*	[74]
143	Cynawilfoside G	*CW*	[74]
144	Cynawilfoside H	*CW*	[74]
145	Cynawilfoside I	*CW*	[74]
146	Gagaminin-3-*O-*β-d-cymaropyranosyl-(1→4)-β-Doleandropyranosyl-(1→4)-β-d-cymaropyranosyl-(1→4)-β-d–cymaropyranosid	*CW*	[95]
147	Lneolon	*CW*	[96]
148	Saccatol D	*CA*	[97]
149	Saccatol E	*CA*	[97]
150	Saccatol F	*CA*	[97]
151	Saccatol G	*CA*	[97]
152	Saccatol H	*CA*	[97]
153	Saccatol I	*CA*	[97]
154	Saccatol J	*CA*	[97]
155	Saccato K	*CA*	[97]
156	Cynsaccatol I	*CA*	[97]
157	Cynsaccatol J	*CA*	[97]
158	Cynsaccatol K	*CA*	[97]
159	Cynsaccatol L	*CA*	[97]
160	Cynsaccatol M	*CA*	[97]
161	Cynsaccatol N	*CA*	[97]
162	Cynsaccatol O	*CA*	[97]
163	Cynsaccatol P	*CA*	[97]
164	Cynsaccatol Q	*CA*	[97]
165	Cynsaccatol R	*CA*	[97]
166	Cynsaccatol S	*CA*	[97]
167	Cynsaccatol T	*CA*	[97]
168	Cynsaccatol U	*CA*	[97]
169	Cynsaccatol V	*CA*	[97]
170	Cynsaccatol W	*CA*	[97]
171	Gagamine	*CA*	[98]

**Table 2:** Acetophenone compounds isolated from *CA*, *CB* and *CW*.

Number	Compounds	Species	References
172	2,4-Dihydroxyacetophenone	*CA CB CW*	[24,28]
173	2,5-Dihydroxyacetophenone	*CA CB CW*	[24,28]
174	4-Hydroxyacetophenone	*CA CB CW*	[24,28]
175	Cynandione A	*CA CW*	[28,30]
176	Cynandione B	*CA CW*	[39]
177	Cynandione E	*CA CW*	[39]
178	Baishouwubenzophenone	*CA CB CW*	[24,29]
179	Cynwilforone A	*CW*	[99]
180	Cynwilforone B	*CW*	[99]
181	Cynwilforone C	*CW*	[99]
182	2-*O*-β-laminaribiosyl-4-hydroxyacetophenone	*CB CW*	[42,43]
183	Bungeiside-C	*CB CW*	[42,43]
184	Bungeiside-D	*CB CW*	[42,43]
185	(+) Cynwilforone D	*CW*	[100]
186	(-) Cynwilforone D	*CW*	[100]
187	(+) Cynwilforone E	*CW*	[100]
188	(-) Cynwilforone E	*CW*	[100]
189	(+) Cynwilforone F	*CW*	[100]
190	(-) Cynwilforone F	*CW*	[100]
191	Cynwilforone G	*CW*	[100]
192	Bungeiside-A	*CB*	[101]
193	Bungeiside-B	*CB*	[101]
194	Picein	*CA*	[15]
195	4′-Hydroxy-3′-methoxyacetophenone	*CW*	[40]
196	1-(2-Hydroxy-4,5-dimethoxyphenyl) ethanone	*CA*	[102]
197	Cynanchone A	*CW*	[103]
198	2,4-Dihydroxy-5-methoxyacetophenone	*CA*	[104]
199	Cynantetrone	*CA*	[105]

**Table 3:** Terpenoids compounds isolated from *CA*, *CB*, and *CW*.

Number	Compounds	Species	References
200	β-sitosterol	*CA*	[38]
201	β-amyrin acetate	*CA*	[38]
202	Wilfolides A	*CW*	[74]
203	Wilfolides B	*CW*	[74]
204	Cycloartenol	*CA*	[13]
205	28α-Homo-β-amyrin acetate	*CA*	[13]
206	11α, 12α-Epoxytaraxer-14-en-3β-yl-acetate	*CA*	[13]
207	δ-Amyrine acetate	*CA*	[13]
208	Taraxaslero acetate	*CA*	[30]
209	Betulinic acid	*CA*	[30]
210	Oleanolic acid	*CA*	[106]
211	Lupeol	*CB*	[107]

**Table 4:** Alkaloids compounds isolated from *CA*, *CB*, and *CW*.

Number	Compounds	Species	References
212	3-Hydroxypyridine	*CA*	[15]
213	3-Hydroxy-2-methylpyridine	*CA*	[15]
214	2-Pyridinemethanol, 5-hydroxy	*CA*	[15]
215	1H-imidazole-5-carboxylic acid	*CA*	[13]
216	6-[(β-d-xylopyranosyl) methyl]-3-pyridinol	*CA*	[16]
217	2-Methyl-6-(2′,3′,4′-trihydroxybutyl)-pyrazine	*CA*	[15]

**Table 5:** Other compounds isolated from *CA*, *CB*, and *CW*.

Number	Compounds	Species	References
218	Ferulic acid methylester	*CA*	[38]
219	Islariciresinol	*CA*	[15]
220	Vomifoliol	*CA*	[15]
221	*n*-Hexacos-5,8,11-trienoic acid	*CA*	[13]
222	Isocopoletin	*CA*	[15]
223	Isofraxidin	*CA*	[15]
224	Adenosine	*CA*	[15]
225	(+)-isolariciresinol	*CA*	[15]
226	4,4-dimethyl heptanedioic acid	*CA*	[15]
227	Leucanthemitol	*CA CW*	[5,41]
228	Suceinie acid	*CA CW*	[5,41]
229	Sucrose	*CA CW*	[38,40]
230	Methyleugenol	*CA CW*	[5]
231	Conduritol F	*CW*	[5]
232	3-(β-d-ribofuranosyl)-2,3-dihydro-6*H*-1,3-oxazine-2,6-dione	*CW*	[14]

**Table 6:** Pharmacological effects of bioactive compounds and extracts isolated from *CA*, *CB* and *CW*.

Number	Bioactive Constituents and Parts	Species	Pharmacology	References
1	Caudatin	*CW* *CA* *CA* *CB* *CB* *CA*	Anti-hepatocellular carcinoma Anti-breast cancer Anti-uterine cancer Anti-human glioma Anti-gastric cancer Anti-lung cancer	[136] [110] [62] [111] [112] [115]
2	Cynandione A	*CA* *CW* *CW* *CW* *CW* *CW*	Neuroprotection Hepatoprotection Anti-inflammatory Reducing liver lipid Reducing blood lipid Hypoglycemic	[57] [58] [137] [70] [71] [99]
3	Wilfoside KIN	*CW* *CW* *CA*	Anti-angiogenic Anti-epileptic Appetite suppression	[130] [74] [37]
4	Aauriculoside A	*CA*	Anti-breast cancer	[93]
5	Caudatin-2,6-dideoxy-3-*O*-methy-β-d-cymaropyranoside	*CA*	Anti-hepatocellular carcinoma	[92]
6	Kidjoranin-3-*O*-α-diginopyranosyl-(1→4)-β-cymaropyranoside	*CA*	Anti-cervical cancer Anti-hepatocellular carcinoma Anti-breast cancer	[64]
7	Kidjoranin-3-*O*-β-digitoxopyranoside	*CA*	Anti-cervical cancer Anti-hepatocellular carcinoma Anti-breast cancer	[64]
8	Caudatin-3-*O*-β-cymaropyranoside	*CA*	Anti-cervical cancer Anti-hepatocellular carcinoma Anti-breast cancer	[64]
9	Gagaminine	*CW*	Antioxidant	[63]
10	Cynandione B	*CA*	Antioxidant	[105]
11	Cynandione E	*CA*	Antioxidant	[105]
12	Cynanchone A	*CA*	Antioxidant	[105]
13	Cynantetrone	*CA*	Antioxidant	[105]
14	Cynanbungeigenin C	*CB*	Anti-human glioma	[91]
15	Cynanbungeigenin D	*CB*	Anti-human glioma	[91]
16	Caudatin-3-*O*-β-d-cymaropyranosyl-(1→4)-β-d-oleandropyranosyl-(1→4)-β-d-cymaropyranosyl-(1→4)-β-d-cymaropyranoside	*CA*	Anti-gastric cancer	[113]
17	Cynsaccatol Q	*CA*	Neuroprotection	[90]
18	Saccatol K	*CA*	Neuroprotection	[90]
19	Cynsaccatol I	*CA*	Neuroprotection	[97]
20	Cynsaccatol N	*CA*	Neuroprotection	[97]
21	Cynsaccatol O	*CA*	Neuroprotection	[97]
22	Cynsaccatol S	*CA*	Neuroprotection	[97]
23	(+) Cynwiforones F	*CW*	Neuroprotection	[100]
24	(−) Cynwiforones F	*CW*	Neuroprotection	[100]
25	Wilfoside C3N	*CA*	Anti-esophageal cancer	[114]
26	Wilfoside C1N	*CW* *CB* *CW* *CA*	Antifungal Immunoregulation Anti-epileptic Appetite suppression	[26] [86] [74] [37]
27	Wilfoside C1G	*CW*	Antifungal	[26]
28	Wilfoside C1GG	*CW*	Antifungal	[26]
29	Cynanauriculoside C	*CA*	Antidepressant	[27]
30	Cynanauriculoside D	*CA*	Antidepressant	[27]
31	Cynanauriculoside E	*CA*	Antidepressant	[27]
32	Otophylloside L	*CA*	Antidepressant	[27]
33	Cynauricuoside C	*CA*	Antidepressant	[27]
34	Cynabungoside A	*CB*	Immunoregulation	[86]
35	Cynabungoside B	*CB*	Immunoregulation	[86]
36	Cynabungoside C	*CB*	Immunoregulation	[86]
37	12-*O*-Nicotinoylsarcostin-3-*O*-β-lcymaropyranosyl -(1→4)-β-d-cymaropyranosyl-(1→4)-α-l-diginopyranosyl-(1→4)-β-d-cymaropyranoside	*CB*	Immunoregulation	[86]
38	Deacylmetaplexigenin-3-*O*-α-cymaropyranosyl-(1→4) -β-cymaropyranosyl-(1→4)-α-cymaropyranosyl-(1→4)-β-cymaropyranosyl-(1→4)-β-cymaropyranoside	*CB*	Immunoregulation	[86]
39	Cynabungone	*CB*	Immunoregulation	[86]
40	Cynabungolide	*CB*	Immunoregulation	[86]
41	2,5-Dihydroxyacetophenone	*CB* *CW*	Skin protection Reducing blood lipid	[121] [71]
42	4-Hydroxyacetophenone	*CW*	Anti-inflammatory	[125]
43	20-*O*-Salicyl-kidjoranin	*CW*	Anti-leukemic	[32]
44	Qingyangshengenin	*CW*	Anti-leukemic	[32]
45	Rostratamin	*CW*	Anti-leukemic	[32]
46	Cynwilforone A	*CW*	Hypoglycemic	[99]
47	Cynawilfoside A	*CW*	Anti-epileptic	[74]
48	Cynauricoside A	*CW*	Anti-epileptic	[74]
49	Cyanoauriculoside G	*CW*	Anti-epileptic	[74]
50	C_21_ steroidal glycoside	*CA* *CA*	Hepatoprotection Anti-inflammatory	[117] [126]
51	Polysaccharides	*CW* *CW* *CA* *CA* *CA*	Anti-inflammatory Antioxidant Menopause suppression Hepatoprotection Immunoregulation	[79] [80] [132] [118] [78]
52	Ethanol extract	*CW CA* *CW* *CW CA CB* *CW* *CW*	Anti-hepatocellular carcinoma Reducing blood lipid Gastric protection Reducing liver lipid Antiviral effect	[25,109] [123] [119] [122] [129]
53	Water extract	*CW* *CW* *CW*	Anti-prostatic Hyperplasia Aphrodisiac Bone-strengthening	[133] [131] [134]
54	Methanol extract	*CA*	Antidepressant activity	[127]

## Data Availability

Not applicable.

## Sample Availability

Samples of the compounds in this paper are available from the authors.

## Abbreviations

UV: Ultraviolet spectrograph, IR: Infrared spectrograph, HPLC: High Performance Liquid Chromatography, MS: Mass Spectrum, NMR: Nuclear Magnetic Resonance Spectrometer PCR: Polymerase Chain Reaction, BLAST: Basic Local Alignment Search Tool, ABTS: 2,2′-Azinobis-(3-ethylbenzthiazoline-6-sulphonate), DPPH: 1,1-Diphenyl-2-picrylhydrazyl radical 2,2-Diphenyl-1-(2,4,6-trinitrophenyl)hydrazyl, TNF-α: Tumor necrosis factor alpha, IL: Interleukin, NF-κB: Nuclear Factor kappa-B, MAPK: Mitogen-Activated Protein Kinase, VEGF: Vascular Endothelial Growth Factor, VCAM: Vascular Cell Adhesion Molecule, LPS: Lipopolysaccharide, LC: Liquid Chromatography, LDL: Low-Density Lipoprotein Cholesterol, HDL: High-density lipoprotein cholesterol.
